# Activation of NF-κB/p65 Facilitates Early Chondrogenic Differentiation during Endochondral Ossification

**DOI:** 10.1371/journal.pone.0033467

**Published:** 2012-03-12

**Authors:** Marjolein M. J. Caron, Pieter J. Emans, Don A. M. Surtel, Andy Cremers, Jan Willem Voncken, Tim J. M. Welting, Lodewijk W. van Rhijn

**Affiliations:** 1 Department of Orthopaedic Surgery, Caphri School for Public Health and Primary Care, Maastricht University Medical Center, Maastricht, the Netherlands; 2 Department of Molecular Genetics, GROW School for Oncology and Developmental Biology, Maastricht University Medical Center, Maastricht, the Netherlands; University of Western Ontario, Canada

## Abstract

**Background:**

NF-κB/p65 has been reported to be involved in regulation of chondrogenic differentiation. However, its function in relation to key chondrogenic factor Sox9 and onset of chondrogenesis during endochondral ossification is poorly understood. We hypothesized that the early onset of chondrogenic differentiation is initiated by transient NF-κB/p65 signaling.

**Methodology/Principal Findings:**

The role of NF-κB/p65 in early chondrogenesis was investigated in different *in vitro*, *ex vivo* and *in vivo* endochondral models: ATDC5 cells, hBMSCs, chicken periosteal explants and growth plates of 6 weeks old mice. NF-κB/p65 activation was manipulated using pharmacological inhibitors, RNAi and activating agents. Gene expression and protein expression analysis, and (immuno)histochemical stainings were employed to determine the role of NF-κB/p65 in the chondrogenic phase of endochondral development. Our data show that chondrogenic differentiation is facilitated by early transient activation of NF-κB/p65. NF-κB/p65-mediated signaling determines early expression of Sox9 and facilitates the subsequent chondrogenic differentiation programming by signaling through key chondrogenic pathways.

**Conclusions/Significance:**

The presented data demonstrate that NF-κB/p65 signaling, as well as its intensity and timing, represents one of the transcriptional regulatory mechanisms of the chondrogenic developmental program of chondroprogenitor cells during endochondral ossification. Importantly, these results provide novel possibilities to improve the success of cartilage and bone regenerative techniques.

## Introduction

Chondrogenic differentiation encompasses the commitment and differentiation of chondro-progenitor cells to chondrocytes. In addition to providing articulating joint surfaces with functional cartilage and maintaining cartilage integrity, chondrogenic differentiation plays an essential role during endochondral ossification. Skeletal growth and bone fracture healing depend on endochondral ossification; growth plate chondrocytes or fracture callus chondrocytes originating from mesenchymal progenitors gradually differentiate into mineralized hypertrophic chondrocytes and finally die by apoptosis. The remaining mineralized extracellular matrix provides a molecular scaffold for infiltrating osteoblasts and osteoclasts to adhere to and remodel, setting the stage for *de novo* bone deposition [1], [2], [3].

Transcriptional targets of NF-κB (nuclear factor kappa-light-chain-enhancer of activated B cells) have been recognized as key developmental signaling mediators that regulate endochondral ossification. Early bone fracture healing by endochondral ossification depends on a haematoma-induced inflammatory environment [4] and several NF-κB-target genes (e.g. interleukin (IL)-6, tumor necrosis factor alpha (TNFα), cyclooxygenase (COX)2 and inducible nitric oxide synthase (iNOS)) are involved in bone fracture repair [5], [6]. Besides its functions in transcriptional regulation of general catabolic inflammatory processes, NF-κB has been linked to skeletal development [7]. Double KO of NF-κB subunits p50 and p52 shows abnormal skeletal development in mice, which was attributed to impaired growth plate function [8]. Recently, NF-κB subunit RelA (p65) was reported to be activated by Nkx3.2 (Bapx1) to control chondrocyte viability [9]. Moreover, RelA was identified as a transcription factor for bone morphogenic protein (BMP)2 [8], [10] and Sox9 (SRY (sex determining region Y)-box 9) in mature chondrocytes during endochondral ossification [11]. Sox9 is expressed by chondroprogenitor cells and is indispensable for chondrogenic differentiation [12], [13], [14]. Sox9 drives the expression of cartilage matrix genes Collagen type II (Col2A1) and Aggrecan cooperatively with L-Sox5 and Sox6 [15], [16], [17] and as such maintains chondrocyte phenotype. The involvement NF-κB/p65 as indispensable factor during chondrogenic development has been studied in the context of mature chondrocytes. However, the mechanisms by which NF-κB/p65 signaling influences early differentiation of chondroprogenitors remains elusive. We hypothesized that the initiation of chondrogenic differentiation is regulated by transient NFκB/p65 signaling. Our data show that during the very first hours of chondroprogenitor differentiation a transient activation of NF-κB/p65 occurs which, in part, regulates the transient expression of key chondrogenic controller Sox9 at the early phase of chondrogenesis. This early transient Sox9 induction precedes the induction of Sox9 that is described to be related to late cartilage matrix synthesis [15], [16], revealing a novel bi-phasic induction for Sox9 during chondrogenic differentiation. We found indications that through the early Sox9 induction the transient NF-κB/p65 activation determines, at least in part, the late stage fate of the chondrogenic differentiation process. Inhibition of NF-κB/p65 mediated signaling is accompanied by inhibition of early Sox9 expression and subsequent inhibition of late stage chondrogenesis. In line with these findings, brief early NF-κB stimulation using different NF-κB activating molecules (LPS, TNFα or BMP2), enhanced chondrogenesis in our *in vitro* and *ex vivo* endochondral models. Our findings demonstrate that NF-κB/p65 signaling, as well as its intensity and timing, is an important factor in the transcriptional regulation of the early chondrogenic developmental program of chondroprogenitor cells and thereby in part determines endochondral ossification.

## Results

### Early ATDC5 differentiation is accompanied by a transient activation of NF-κB/p65 and expression of Sox9

**Figure 1:**
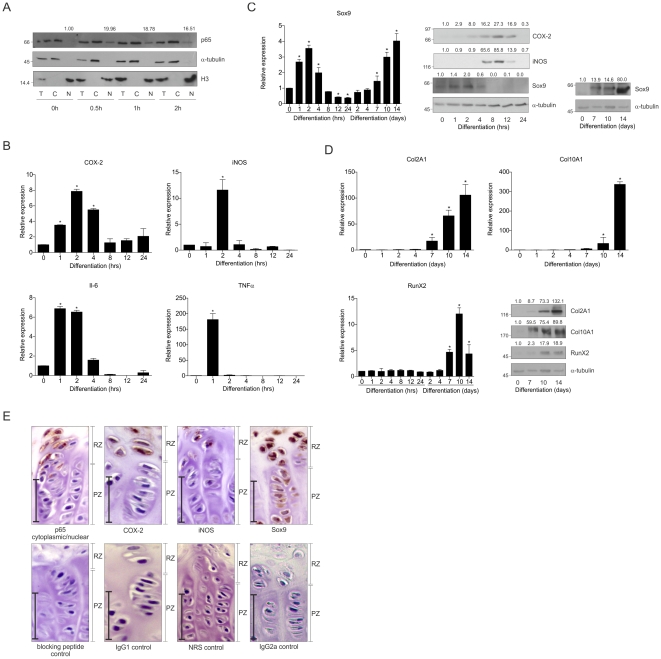
Early chondrogenic differentiation is accompanied by NF-κB/p65 activation and transient Sox9 expression. **A:** Nuclear translocation of the NF-κB subunit p65. Total extract (T), cytoplasmic (C) and nuclear (N) fractions were isolated at 0, 0.5, 1 and 2 hours in ATDC5 differentiation. Cytoplasmic marker: α-tubulin, nuclear marker: Histone H3. **B:** COX-2, iNOS, Il-6 and TNFα mRNA expression at 0–24 hours in differentiation (relative to t = 0 and corrected for β-actin). **C:** Sox9 mRNA expression during chondrogenic differentiation (left panel). Protein expression of Sox9, COX-2 and iNOS at 0–24 hours (middle panel) and for Sox9 also at 0, 7, 10 and 14 days in differentiation (right panels). Molecular weight markers (kDa) are depicted on the left of immunoblots and relative quantifications are depicted on top of immunoblots. * = p<0.05. **D:** mRNA and protein expression of Col2A1, Col10A1, RunX2 during ATDC5 differentiation **E:** Sections from 6 weeks old mouse growth plates (resting (RZ) and proliferative (PZ) zones) stained for p65, COX-2, iNOS and Sox9. Lower panels show appropriate negative controls. Bars = 50 µm.

As a model for endochondral ossification, the murine chondroprogenitor ATDC5 cell line was used [18], [19]. Early involvement of NF-κB/p65 signaling was assessed by examining subcellular localization of the NF-κB subunit p65 [20] (Figure 1A). In proliferating cells (t = 0) p65 was not detectable in the nucleus. However, upon initiation of chondrogenesis a fraction of cytoplasmically localized p65 translocated to the nucleus, which was readily detectable at 30 minutes post-induction of differentiation. The nuclear occupation of p65 peaked between 0.5 and 4 hours and was not detectable anymore after 8 hours (Figure S1). To further verify overall activation of NF-κB/p65, expression of NF-κB-target genes was measured (Figure 1B). Induction of COX-2, iNOS, Il-6 and TNFα mRNAs was detectable between 1 and 4 hours in differentiation and returned to baseline levels around 8 hours. COX-2 and iNOS proteins showed a similar transient expression (Figure 1C). To verify potential cross-talk between the differentiation program and the early NF-κB/p65 response, expression of Sox9 was determined. During ATDC5 differentiation Sox9 expression was transiently induced at 1–4 hours in differentiation and steadily increased again from day 7 in differentiation (Figure 1C). Remarkably, these data imply that the expression of Sox9 during chondrogenic differentiation in ATDC5 is bi-phasic. Confirming completion of the chondrogenic differentiation program in the ATDC5 cells chondrogenic markers Col2A1, Col10A1, and RunX2 increased in expression from day 7 in differentiation (Figure 1D).

Previously it was reported that an NF-κB/p65 transcription factor binding site is located in the Sox9 gene [11]. *In silico* screening of Sox9 promoter regions detected two other putative evolutionary conserved NF-κB/p65 transcription factor binding sites in various mammals (Figure S2).

Post-natal growth plates contain a pool of dedicated chondroprogenitor cells in the so-called resting zone. During growth plate development, these resting zone cells differentiate into proliferating chondrocytes and are thus responsible for cartilage generation in the growth plate [21]. To verify whether activation of NF-κB/p65 can also be detected in early chondrogenesis during endochondral ossification *in vivo*, the resting- and proliferative zones of 6 week old mice growth plates were analyzed for expression of Sox9, p65 and NF-κB/p65-target genes (COX-2 and iNOS) (Figure 1E). As described previously [22], Sox9 expression was detected in the resting zone (RZ) cells as well as in proliferative zone chondrocytes (PZ). Interestingly, Sox9 seems to be more abundantly expressed in the resting zone. Expression of p65 was found in the cytoplasm of RZ cells and was not detectable in the PZ. Also, p65 was found to be localized in the nuclei of several RZ cells. Both the expression of the NF-κB/p65-target genes COX-2 and iNOS was found in the RZ.

Overall, these results indicate that an NF-κB/p65 signaling response occurs very early in chondrogenic differentiation and correlates with a thusfar unknown early transient induction of Sox9 in ATDC5 cells.

### Inhibition of early NF-κB/p65 activation leads to impaired chondrogenic differentiation

**Figure 2:**
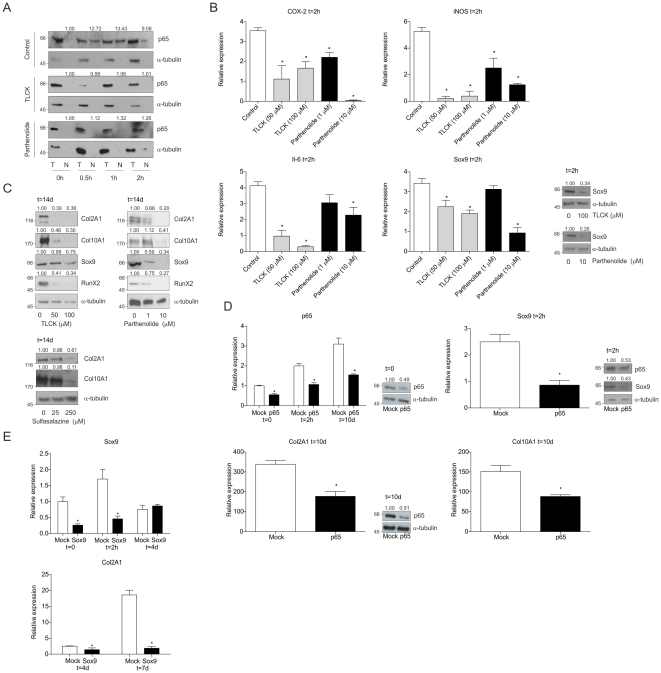
Inhibition of early NF-κB/p65-activation leads to impaired ATDC5 differentiation. **A:** Cells were differentiated in the absence (control) or presence of TLCK (100 µM) or Parthenolide (10 µM) and total (T) and nuclear (N) fractions were prepared. NF-κB was detected as p65. **B:** Expression of COX-2, iNOS, Il-6 and Sox9 mRNAs at 2 hours in differentiation in the presence of TLCK or Parthenolide. Sox9 protein expression was determined from similar samples (right panel set). **C:** Protein expression at day 14 in differentiation of Col2A1, Col10A1, Sox9 and RunX2 with TLCK (left panels), Parthenolide (right panels) or Sulfasalazine (lower left panels). **D:** Knock-down (KD) of p65 mRNA and protein at 0, 2 hours and 10 days in differentiation (“Mock” below figures indicates scrambled siRNA and “p65” below figures indicates p65 KD). Upper right set: expression of Sox9 mRNA and protein in p65 KD cells at 2 hours in differentiation. Lower sets: messenger RNA expression of Col2A1 and Col10A1 at 10 days in differentiation of p65 KD cells. * = p<0.05. **E:** Sox9 mRNA expression at 0, 2 hours and 4 days in differentiation of cells transfected one day prior to differentiation with scrambled (indicated by “Mock”) or Sox9 siRNA (indicated by “Sox9”) (upper panel). Col2A1 mRNA expression was determined at day 4 and 7 in differentiation (lower panel). * = p<0.05.

To functionally determine the role of the early NF-κB/p65 activation in relation to initiation of chondrogenic differentiation, nuclear translocation of NF-κB was inhibited by TLCK or Parthenolide. Dose-response experiments (data not shown) revealed optimal inhibitor concentrations: 100 µM TLCK or 10 µM Parthenolide efficiently inhibited NF-κB nuclear translocation during early ATDC5 chondrogenesis (Figure 2A). In agreement with efficient NF-κB inhibition, both TLCK and Parthenolide inhibited COX-2, iNOS and Il-6 mRNA expression at 2 hours in differentiation (Figure 2B). We next tested the functional relationship between the early NF-κB/p65 response and chondrogenic differentiation. Early Sox9 levels were reduced by both TLCK and Parthenolide (Figure 2B and Figure S3A). Inhibition of NF-κB activation by TLCK or Parthenolide resulted in a similar dose-dependent inhibition of late phase (day 14) Col2A1, Col10A1, RunX2 and Sox9 protein expression (Figure 2C). Results were confirmed using the clinically used NF-κB inhibitor Sulfasalazine (Figure 2C). To independently verify the effect of pharmacological NF-κB/p65 inhibition on Sox9 expression during early chondrogenic differentiation, we genetically targeted NF-κB/p65 by RNAi. Transient transfection of a p65 siRNA duplex in ATDC5 reduced p65 mRNA and protein expression at 2 hours in differentiation by ∼50% (Figure 2D, upper left set). Expression of COX-2, iNOS and Il-6 were also reduced as compared to Mock transfection (data not shown). In good agreement with the results described above, early expression of Sox9 mRNA and protein was significantly reduced by p65 knock-down (Figure 2D, upper right set). To further validate how p65 knock-down affects long term chondrogenic differentiation, p65 mRNA expression was targeted for a longer timeframe by re-transfection of the siRNA duplex. We confirmed efficient knock-down of p65 mRNA at 10 days in differentiation (Figure 2D, upper left and lower left sets). Functionally the p65 knock-down resulted in impaired chondrogenic differentiation (Figure 2D, lower left and lower right). Finally, to test whether the early transient Sox9 induction influences late chondrogenic differentiation we targeted the expression of Sox9 only in the early phase of differentiation by one single Sox9 siRNA transfection, followed by 7 days of differentiation follow-up under normal differentiation conditions. Efficient knock-down of Sox9 mRNA was confirmed at the start of differentiation. At 2 hours in differentiation the early induction of Sox9 was almost completely abolished (Figure 2E, upper panel). At day 4, Sox9 siRNA treatment was not effective anymore, as no difference in Sox9 expression was detectable between conditions. To verify the consequence of this early Sox9 knock-down, the expression of Col2A1 was determined (Figure 2E, lower panel). Col2A1 expression was detectable from day 4 on. At day 4 a significant difference was found between control and the early Sox9 knock-down. This difference was even bigger at day 7, where early Sox9 knock-down lead to an almost abolished expression of Col2A1. Taken together, these results indicate that inhibition of NF-κB/p65 nuclear translocation suppresses the initiation of chondrogenic differentiation, by inhibiting early Sox9 induction and subsequent expression of late chondrogenic markers. This indicates that late phase chondrogenic development and endochondral ossification are, at least in part, regulated by early NF-κB/p65 and Sox9 signaling events.

### Late phase ATDC5 differentiation is enhanced by stimulation of early NF-κB/p65 activity

**Figure 3:**
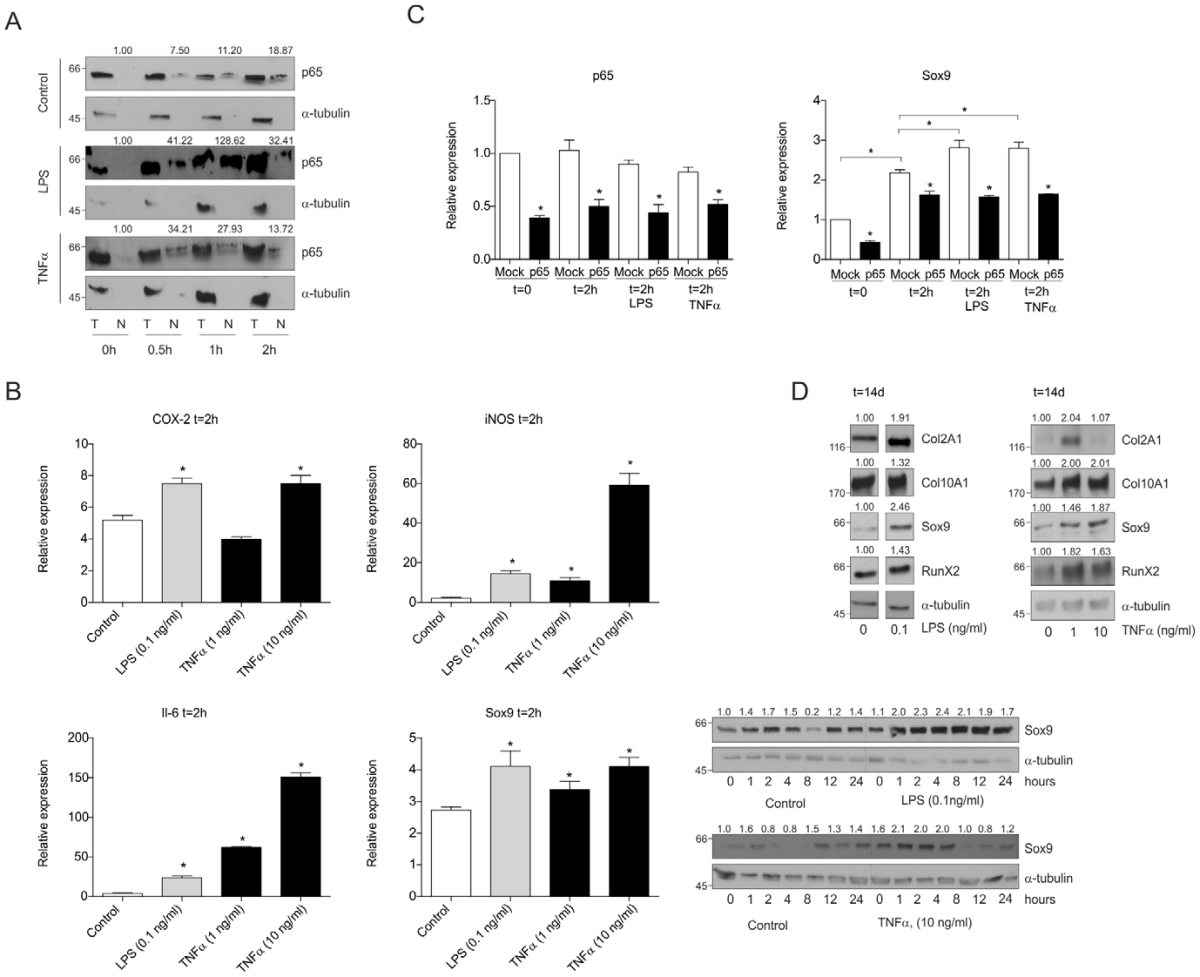
Late phase ATDC5 differentiation is enhanced by stimulation of early NF-κB/p65 activity. **A:** Cells were differentiated in the absence (control) or presence of LPS (0.1 ng/ml) or TNFα (10 ng/ml) and total (T) and nuclear (N) fractions were prepared. NF-κB was detected as p65. **B:** Expression of COX-2, iNOS, Il-6 and Sox9 mRNAs at 2 hours in differentiation in LPS or TNFα treated cells. Far right panel set: early Sox9 protein expression in LPS or TNFα treated cells. **C:** ATDC5 cells were transfected with a p65 siRNA duplex or scrambled (“Mock”) siRNA duplex and differentiated in the absence or presence of LPS (0.1 ng/ml) or TNFα (10 ng/ml) for 2 hours. Knock-down of p65 mRNA was confirmed at 0 and 2 hours in differentiation (left panel). Right panel shows Sox9 mRNA expression. **D:** Protein expression of Col2A1, Col10A1, Sox9 and RunX2 in differentiated ATDC5 cells (14 days) in the presence of LPS (left panel set) or TNFα (right panel set), only during the first 24 hours of differentiation. * = p<0.05.

The association between early NF-κB/p65 activation and initiation of chondrogenic differentiation prompted us to test whether stimulation of NF-κB/p65 enhances chondrogenic differentiation. We enforced NF-κB/p65 signaling by supplementing differentiation medium with the NF-κB/p65-activating molecular tools LPS or TNFα. These activating agents were added to the differentiating culture during the first 24 hours of differentiation only (the timeframe in which the early NF-κB/p65 activation takes place). At low LPS concentrations (0.1 ng/ml) and TNFα (10 ng/ml), NF-κB/p65 nuclear translocation was slightly enhanced during early chondrogenic differentiation (Figure 3A) and resulted in increased early expression of COX-2, Il-6 and iNOS mRNAs (Figure 3B). Interestingly, increasing NF-κB/p65 activity enhanced the magnitude of transient Sox9 (as well as L-Sox5 and Sox6) expression early in chondrogenic differentiation (Figure 3B and Figure S3A). Interference with NF-κB-signaling by siRNA mediated p65 knock-down attenuated the LPS- and TNFα-induced increased Sox9 expression back to Mock-treated differentiation levels (Figure 3C). Moreover, increased Col2A1, Col10A1, Sox9 and RunX2 expression in 24 hour LPS- and TNFα-exposed cells was detected at day 10 and 14 in differentiation (Figure 3D and Figure S3B). Data show that early and short chondrogenic NF-κB/p65-activation positively responds to environmental stimulation of NF-κB/p65, resulting in overall increased chondrogenic potential late in differentiation.

### Early transient NF-κB/p65 signaling during endochondral differentiation of primary mesenchymal progenitor cells

**Figure 4:**
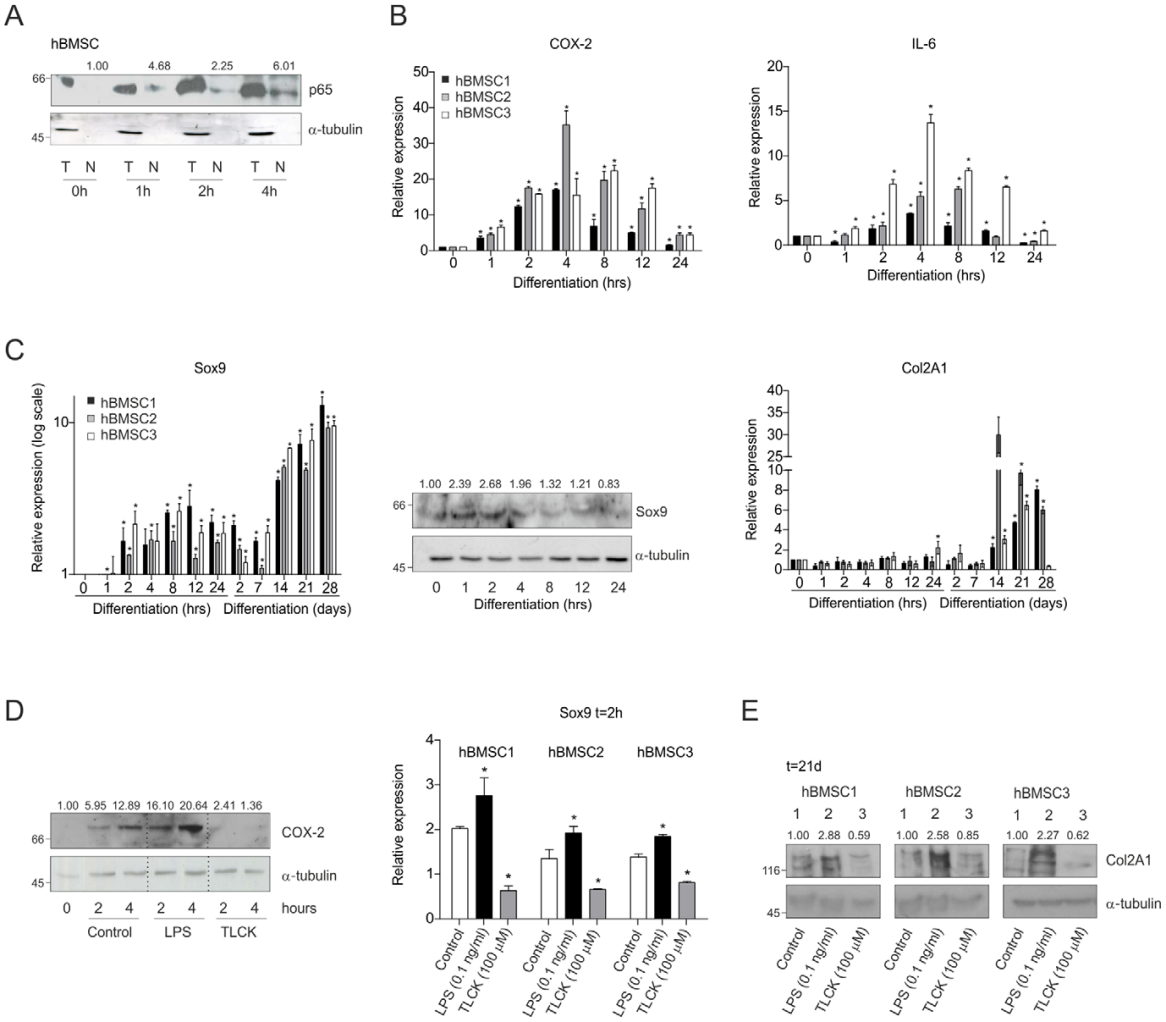
Transient NF-κB/p65 signaling during early chondrogenic differentiation of human bone marrow stem cells. Human bone marrow stem cells from three individuals (hBMSC1/2/3) were differentiated into the chondrocyte lineage using monolayer culture. **A:** Nuclear (N) and total (T) fractions were isolated from 0, 1, 2, 4 hours samples. NF-κB was detected as p65, cytoplasmic marker: α-tubulin. **B:** Expression of COX-2 and IL-6 mRNAs at 0–24 hours in hBMSC differentiation. **C:** Left; Sox9 mRNA expression during hBMSC differentiation, Sox9 protein expression during 0–24 hours in hBMSC differentiation (middle) and Col2A1 mRNA expression during hBMSC differentiation (right). **D:** Left; COX-2 protein expression at 0, 2, 4 hours in differentiation in the presence of LPS (0.1 ng/ml) or TLCK (100 µM). Right; Sox9 mRNA expression at 4 hours in differentiation in the presence of LPS (black bars) or TLCK (grey bars). **E:** Col2A1 protein expression in day 21-samples of differentiated hBMSCs. Lanes 1: control condition, Lanes 2: 0.1 ng/ml LPS (only first 24 hours) and lanes 3: 100 µM TLCK. * = p<0.05.

To verify the results obtained with the ATDC5 system, similar endochondral differentiation experiments were performed using human bone marrow stem cells (hBMSC). In hBMSCs, Col2A1 and Col10A1 mRNA and protein expression was evident from day 7–21 in chondrogenic differentiation (Figure 4C and Figure S4A). Expression of Col1A1 and PPARγ was not upregulated, ruling out osteogenic or adipogenic differentiation from this multipotent cell source during chondrogenic induction (data not shown). In concordance with ATDC5, p65 translocated to the nucleus at 1 hour in hBMSC differentiation and nuclear p65 levels further sustained up to 4 hours (Figure 4A). Analyses showed that hBMSC chondrogenic differentiation of 3 independent isolates is accompanied by transient expression of COX-2 and IL-6 early in differentiation (Figure 4B). Furthermore, as in ATDC5, induction of Sox9 expression in hBMSC differentiation was also bi-phasic: from 1–2 hours onward, as well as late (day 14–28) in differentiation (Figure 4C). In agreement with reduced NF-κB/p65 activity, COX-2, Sox9 and IL-6 expression levels decreased in the presence of TLCK (Figure 4D; COX-2 immunoblot; grey bars in graph and Figure S4B/C). Conversely, brief stimulation of NF-κB/p65 activity by low LPS concentrations during the first 24 hours only, enhanced NF-κB/p65-target gene expression, as well as early Sox9 expression (Figure 4D; COX-2 immunoblot; black bars in graph and Figure S4B/C). Coherent with the murine model, human chondroprogenitor cells clearly showed increased expression of Col2A1 at day 21 when exposed to LPS during the first 24 hours in differentiation (Figure 4E; lanes 2) and lower Col2A1 levels in the presence of TLCK (Figure 4E; lanes 3). Taken together, these data show that hBMSC endochondral differentiation also integrates a transient NF-κB/p65 activation during the early initiation of differentiation, ultimately contributing to the outcome of the chondrogenic cell fate.

### NF-κB/p65 signaling induces chondrogenic marker gene expression in mesenchymal progenitor cells without the addition of chondrogenic growth factors

**Figure 5:**
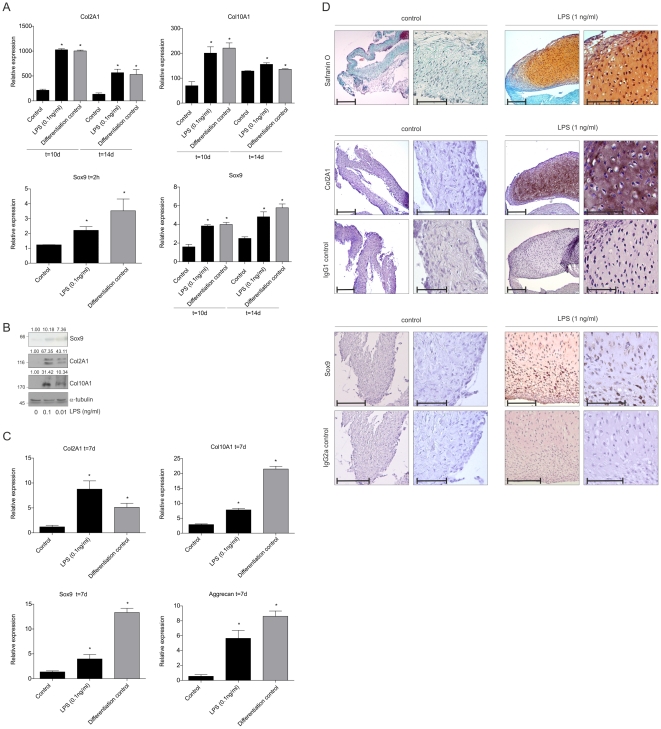
NF-κB/p65 activation induces chondrocyte marker gene expression without the addition of chondrogenic growth factors. **A:** Col2A1, Col10A1 and Sox9 mRNA expression at 2 hours (Sox9 only), 10 and 14 days in ATDC5 cells, cultured in proliferation medium in the absence (“control”) or presence (first 24 hours) of LPS (black bars). Col2A1, Col10A1 and Sox9 mRNA expression of standard differentiated ATDC5 is shown for comparative purposes (grey bars; “differentiation control”). **B:** Sox9, Col2A1 and Col10A1 protein expression in a representative hBMSC sample cultured for 21 days in proliferation medium with 0.1 or 0.01 ng/ml LPS (first 24 hours). **C:** Col2A1, Col10A1, Sox9 and aggrecan mRNA expression in cPACs (chicken Periosteum Agarose Culture) cultured in proliferation medium for 7 days in the absence or presence of LPS during the first 48 hours (black bars). Col2A1, Col10A1, Sox9 and aggrecan mRNA expression of cPACs differentiated in standard differentiation medium (containing TGFβ3 and insulin, see also Materials and Methods) is shown for comparative purposes (grey bars). * = p<0.05. **D:** In similar samples from (C) sections (5 µm) from cPACs were stained by Safranin O/Fast green (upper set), for Col2A1 (middle set) and Sox9 (lower set). For Safranin O and Col2A1 stainings: bars = 200 µm for first and third column micrographs and 100 µm for second and fourth column of micrographs. For Sox9 staining, bars = 150 µm for first and third column of micrographs and 100 µm for second and fourth column of micrographs.

The progenitor cell intrinsic NF-κB/p65 activation occurs as a result of environmental differentiation conditions, but positively responds to NF-κB stimulating agents (LPS and TNFα) early in chondrogenic differentiation. We therefore tested whether an NF-κB/p65-activating stimulus alone would be able to facilitate chondrogenic signaling in mesenchymal progenitors, without the addition of other differentiation factors like insulin or TGFβ3. To this end, ATDC5 proliferation medium was supplemented with only LPS (first 24 hours alone) and subsequently cells were cultured in proliferation medium for 10 or 14 days. A brief stimulation with LPS induced NF-κB/p65 activation (data not shown) and, in contrast to the control condition, resulted in a transient Sox9 expression at 2 hours after LPS exposure (Figure 5A, lower left panel) and equal Sox9, Col2A1 and Col10A1 mRNA expression levels (at day 10 and 14) as normal differentiation conditions do in ATDC5 (Figure 5A). However Col10A1 expression in the proliferation condition at 14 days appeared unexpectedly high as compared to the LPS and differentiation conditions. Briefly, findings were verified in hBMSCs. After 21 days culture in proliferation medium (without insulin and TGFβ3), hBMSCs expressed Sox9 very lowly and Col2A1 and Col10A1 were not expressed (Figure 5B), whereas brief stimulation with LPS (0.1 and 0.01 ng/ml) during the first 24 hours only, resulted in robust expression of Sox9, Col2A1 and Col10A1 protein at 21 days (Figure 5B). To further establish the relevance of our findings in tissue involved in endochondral ossification, we adopted an *ex vivo* periosteal tissue differentiation model for chondrogenesis using chicken embryonal periosteum [23]. Importantly, as a source for mesenchymal progenitor cells, periosteal tissue is directly relevant for endochondral ossification processes and fracture healing. Harvested periosteal tissue from the chicken tibia was cultured between agarose layers (chicken periosteum agarose culture: cPAC). After 1 week of culturing in proliferation medium chicken periosteal explants did not acquire any chondrogenic properties (Figure 5C; black control bars and Figure 5D; left micrographs). In contrast, supplementation of the culture medium with LPS for the first 48 hours only, resulted in the formation of cartilaginous tissue after 1 week, as determined by upregulation of Col2A1, Col10A1, Sox9 and aggrecan mRNA expression (Figure 5C; second black bars), as well as positive Safranin O staining, immunohistochemical detection of Sox9 and Col2A1 as well as typical chondrocyte morphology (Figure 5D). For comparative purposes, same chondrogenic markers were measured in cPACs that were differentiated in standard differentiation medium (containing insulin and TGFβ3) (Figure 5C; grey bars). These data indicate that in mesenchymal progenitor cells a short exogenous NF-κB/p65-activating stimulus may result in cellular signaling through chondrogenic pathways which can explain the expression of chondrocyte marker molecules.

### BMP2 activates NF-κB/p65 in early ATDC5 chondrogenic differentiation

**Figure 6:**
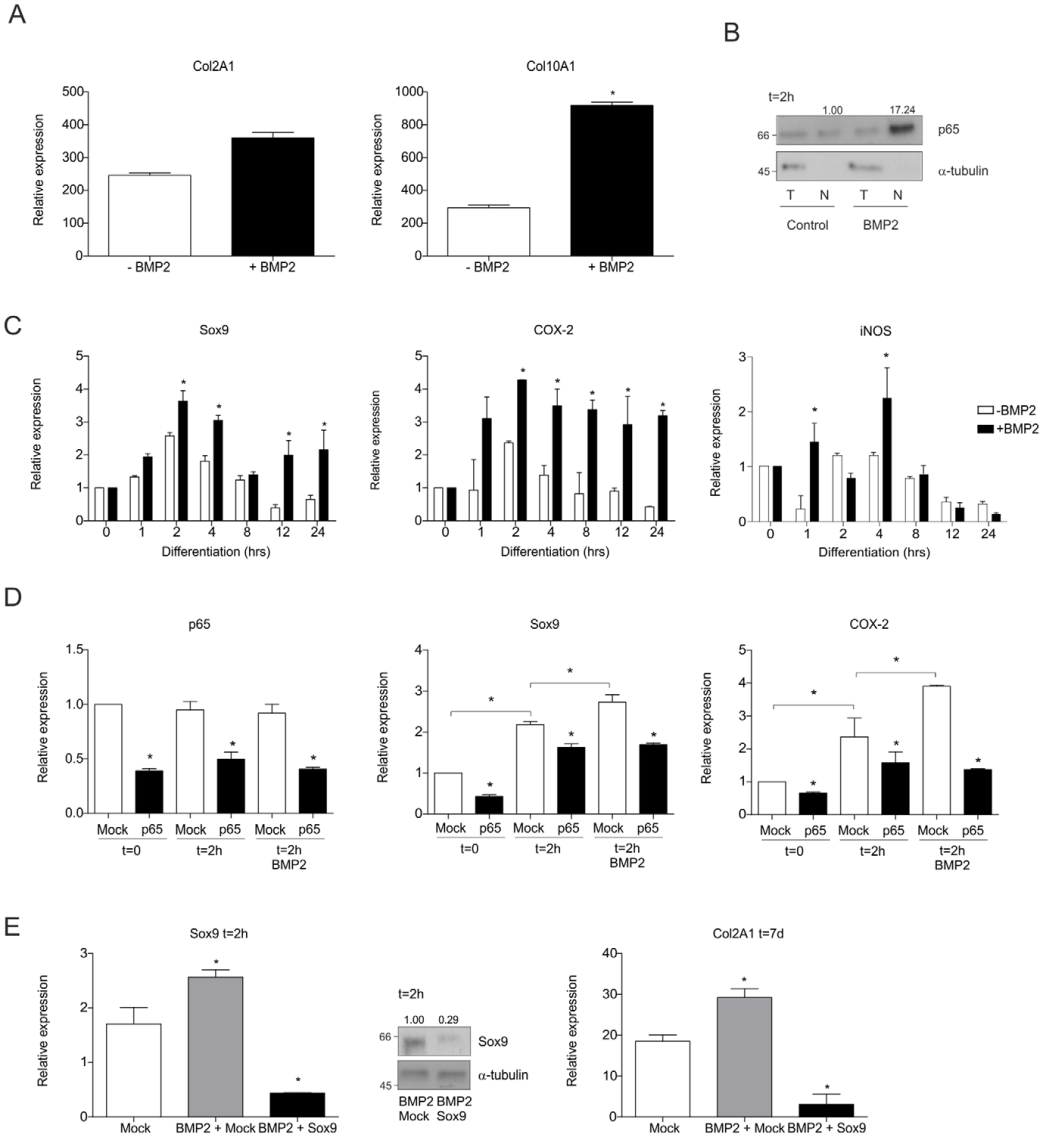
BMP2 activates NF-κB/p65 in early ATDC5 chondrogenic differentiation. **A:** ATDC5 was differentiated for 14 days in the absence or presence of BMP2 (30 ng/ml). Relative mRNA expression of Col2A1 and Col10A1 was determined. **B:** Cells were differentiated in the absence (control) or presence of BMP2 (30 ng/ml) and total (T) and nuclear (N) fractions were prepared at 2 hours in differentiation. NF-κB was detected as p65. **C:** Cells were differentiated for 0–24 hours in the absence or presence of BMP2 and expression of Sox9 (left) and NF-κB-targets COX-2 (middle) and iNOS (right) was determined. **D:** ATDC5 cells were transfected with a p65 siRNA duplex or Mock siRNA duplex and differentiated in the absence or presence of BMP2 for 2 hours. Knock-down of p65 mRNA was confirmed at 0 and 2 hours in differentiation (left). Middle and right graphs show Sox9 and COX-2 mRNA expression, respectively. **E:** Left panel set: Sox9 KD at mRNA and protein level at 2 hours in differentiation in cells transfected with scrambled (indicated as “Mock”) siRNAs, scrambled siRNAs in the presence of BMP2, or Sox9 siRNAs (indicated as “Sox9”) in the presence of BMP2. Transfection was done the day prior to differentiation and BMP2 treatment was for the first 24 hours only. Right panel: Col2A1 mRNA expression was determined in same experiment at day 7 in differentiation. * = p<0.05.

LPS and TNFα were used as tools to activate NF-κB/p65. Exceptions left alone (e.g. TNFα in OA and RA), these activators are not known to be present in the cartilaginous environment. We therefore asked whether growth factors may support the initiation of an early NF-κB/p65 activation in the way described herein. BMP's are known to play crucial roles in early mesenchymal condensation by regulating Sox9 expression [24] and contributing to other phases of the endochondral ossification processes. Also, BMP2 has been described to be able to activate NF-κB/p65 in chondrocytes [25]. As shown in Figure 6A, 30 ng/ml BMP2 resulted in increased expression of Col2A1 and Col10A1 in differentiating ATDC5 cells. To verify whether a similar early NF-κB/p65 activation might involve this BMP2 action, we analyzed p65 nuclear translocation. We found that p65 nuclear translocation at 2 hours in differentiation was more increased in the presence of BMP2 as compared to control (Figure 6B). Increased and more prolonged expression of Sox9, COX-2 and iNOS in the first 24 hours of differentiation confirmed downstream NF-κB/p65-activated pathways (Figure 6C). To further establish a role for p65 in this process, ATDC5 cells were transfected with a p65 siRNA duplex or scrambled siRNA duplex and differentiated in the absence or presence of BMP2 (30 ng/ml) (Figure 6D). Knockdown of p65 mRNA was confirmed at 0 and 2 hours in differentiation (left panel). Middle and right panels show Sox9 and COX-2 mRNA expression, respectively. As described above, Sox9 and COX-2 mRNA expression increased at 2 hours in differentiation and increased further with BMP2 stimulation (see also Figure 6C). The BMP2-initiated increased Sox9 and COX-2 mRNA upregulation is inhibited to equal levels as the differentiated control p65 knock-down condition without BMP2 supplementation, supporting a role for p65 in this mechanism. Finally we addressed whether BMP2 might exert its prochondrogenic action early in differentiation through the NF-κB/p65 induced early Sox9 expression. The early Sox9 mRNA expression was targeted by a single Sox9 siRNA transfection (see also Figure 2E) and differentiation was initiated in the presence of BMP2 (first 24 hours alone). As shown in Figure 6E, in the presence of BMP2, Sox9 siRNA transfection resulted in an efficient knock-down of Sox9 mRNA and protein expression at 2 hours in differentiation. The early knock-down of Sox9 under BMP2 treatment at 2 hours in differentiation resulted in impaired Col2A1 expression at 7 days into differentiation. Overal, these results suggest that BMP2 action in the early chondrogenic phase of endochondral ossification may, in part, be explained via the herein described early transient NF-κB/p65 activation and Sox9 expression. These findings may provide a possible *in vivo* context for the herein described NF-κB/p65 pathway.

## Discussion

We here report that chondrogenic differentiation of chondroprogenitor cells is, at least in part, determined by early activation of NF-κB/p65 which subsequently contributes to the initiation of chondrogenic differentiation by regulating the early expression of key chondrogenic factor Sox9.

Inflammatory mediators play crucial roles in cartilage degenerative conditions such as rheumatoid arthritis and osteoarthritis [26], [27], [28]. Most, if not all of these inflammatory mediators are regulated via activated NF-κB pathways. However, recent studies reported that in chondrocytes NF-κB/p65-target genes are not exclusively associated with cartilage degenerative conditions [29]. TNFα was recently described to regulate expression of BMP2 via an NF-κB/p65 dependent mechanism [8], [30], [31]. Involvement of NF-κB was reported during development of the growth plate [8], [10], as well as in preventing apoptosis of maturating chondrocytes via interaction with Nkx3.2 [9]. NF-κB/p65 has been reported to function as a transcription factor for Sox9 in mature chondrocytes [11] and finally, Aung and colleagues provided evidence that OA chondrocytes excrete soluble factors that initiate chondrogenic differentiation of human mesenchymal stem cells [32]. Except for the last study, most of these previous investigations were limited by the use of maturated chondrocytes, thereby leaving the question at which chondrogenic stage an imperative nuclear NF-κB/p65 presence would be required for differentiation. As we made use of cellular differentiation models that initiate chondrogenic differentiation from a progenitor stage onward, our experiments provided the possibility to address the role of NF-κB/p65 activity in a specific chondrogenic phase, while at the same time determining the consequences of NF-κB/p65 activity during subsequent later chondrogenic phases. We found that Sox9 induction during chondrogenic differentiation of chondroprogenitor cells is bi-phasic and is evident during the first hours of differentiation and induced for a second time later on in differentiation. The late Sox9 induction follows chondrocyte matrix expression and is thereby expected to transcriptionally regulate the induction of cartilage matrix genes such as Col2A1 and aggrecan [15], [16]. In addition to late Sox9 expression, upon early chondrogenic differentiation NF-κB/p65 transiently translocates to the nucleus, thereby enabling NF-κB/p65-driven early Sox9 transcription. Although the function of the novel short Sox9 pulse during the early start of chondrogenesis remains to be elucidated, our data indicate that it might function in the context of the Sox-trio [13], [17] and is important in determining the chondrogenic outcome, possibly by priming the early differentiating cell for chondrogenic commitment by yet unknown (epigenetic) mechanisms.

Next to transcriptional induction of Sox9, the early chondrogenesis-associated NF-κB/p65 activation results in additional transient expression of inflammatory target genes, such as COX-2, iNOS, Il-6 and TNFα. Expression of these inflammatory NF-κB/p65 target genes may be an aspecific result of the transient activation of NF-κB/p65. However ample experimental evidence supports a critical role for these inflammatory mediators in cellular fate determination in the context of the endochondral ossification during fracture healing, as the respective knock-out mice display a severely impaired fracture healing capacity [6], [33], [34], [35], [36], [37], [38], [39], [40], [41], [42]. Human MSCs have been reported to excrete several chondrogenic growth factors (e.g. IGF1 and FGF2) upon inflammatory LPS or TNFα stimulation [43], [44] and our data (Figure S5) show that treatment of differentiating ATDC5 cells with TNFα, for the first 24 hours only, also resulted in significantly higher expression of chondrogenic growth factors (IGF1, TGFβ1, FGF3, BMP2, BMP4) from 7 days on in differentiation. It is therefore tempting to speculate that the expression of NF-κB/p65-targets during the onset of chondrogenic differentiation may have an additional function in the paracrine signaling for later stages during endochondral ossification. Also, despite the degenerative environment, the endochondral formation of cartilaginous osteophytes is a hall mark of OA [45]. An aspect of their formation may also be found in NF-κB/p65-driven chondrogenic differentiation of synovial or periosteal progenitors, initiated from the degenerating OA cartilage. As OA-like conditions are absent in the developing growth plate, expression of NF-κB/p65-targets and growth factors by differentiating growth plate chondrocytes may maintain growth plate chondrogenic differentiation of local resting zone progenitor cells in a similar paracrine fashion.

Although in early chondrogenic differentiation NF-κB/p65 is clearly activated during the first hours in the differentiation process, we do not yet fully understand how the chondrogenic culture environment triggers this inflammatory response. Several chondrogenic growth factors are associated with NF-κB/p65 signaling. It is known that TGFβ-receptor (TGFR) and IGF-receptor (IGFR) signaling activate NF-κB and expression of chondrogenic markers in chondrocytes [46], [47]. Key extracellular signaling molecules triggering chondrogenesis *in vitro* are insulin and TGFβ. Hence, insulin/IGFR- and TGFβ/TGFR-activation likely initiate signaling through NF-κB/p65, resulting in initiation of early transient Sox9 induction. Our observation that stimulation of NF-κB activity by LPS or TNFα under chondrogenic conditions or even under proliferation conditions (in the absence of standard chondrogenic stimuli) enhances or triggers Sox9 expression and eventually contributes to the chondrogenic potential, is well in line with this notion. Although mesenchymal progenitor cells express TNFR and TLR2/4 [48], [49], the use of LPS and TNFα to activate NF-κB might be contradictory in the context of chondrogenic differentiation. However, these agents were solely used as NF-κB-activating tools in the herein described work. More relevant to the *in vivo* context of early chondrogenic differentiation and endochondral ossification BMP2 has previously been described to be able to translocate NF-κB/p65 to the nucleus [25] and to be involved in Sox9 regulation during early mesenchymal condensation [24]. These previous findings may provide an *in vivo* context in which the herein described BMP2 mediated NF-κB/p65 driven early Sox9 expression may function. In addition, other studies [8], [10] have shown that BMP2 expression itself can be regulated by NF-κB/p65 during late chondrogenesis in maturated chondrocytes, thereby contributing to longitudinal bone growth and preventing apoptosis of these chondrocytes. Therefore, our and previous findings indicate that BMP2 action and regulation might depend on the chondrogenic differentiation status [30].

In conclusion, our data indicate that initiation of chondrogenic differentiation during endochondral development, at least in part, depends on an early activation of NF-κB/p65. The early NF-κB/p65 activation evokes a novel early and transient expression of Sox9, which, together with a late Sox9 induction, contributes to the outcome of the chondrogenic differentiation program of mesenchymal progenitor cells. Our findings complement previously reported NF-κB/p65 involvement in chondrogenic differentiation and provide novel insight into the origin, timing and dynamics of NF-κB/p65-induced gene expression in early chondrogenic differentiation. These data add to an emerging and growing concept [7] where differentiating chondrocytes and endochondral development are regulated by NF-κB/p65-mediated processes and may be used as new leads to modulate chondrogenic differentiation in cartilage and bone regenerative medicine approaches such as the ACI technique [50] and the *in vivo* bioreactor technique [51], [52].

## Materials and Methods

### ATDC5 cell culture

ATDC5 cells [18] were cultured in proliferation medium (DMEM/F12 (Invitrogen), 5% FCS (PAA), 1% antibiotic/antimycotic (Invitrogen) and 1% NEAA (Invitrogen)). Differentiation medium comprised proliferation medium supplemented with 10 µg/ml insulin (Sigma, St. Louis, MO, USA), 10 µg/ml transferrin (Roche Applied Science) and 30 nM sodium selenite (Sigma). Cells were plated at 6,400 cells/cm^2^ and the following day chondrogenesis was initiated by changing the proliferation medium to differentiation medium (or proliferation medium in case of data presented in Figure 5). Medium was changed every 2 days and every day from day 10 onwards. To inhibit NF-κB, TLCK (Acros), Parthenolide (Sigma) and Sulfasalazine (Sigma) were used. LPS (Sigma) or TNFα (R&D) were used as NF-κB/p65 activators. BMP2 was used at 30 ng/ml (Sigma). For RNAi-experiments a p65 siRNAduplex (sense: 5′-AGAGGACAUUGAGGUGUAUTT-3′, anti-sense: 5′-AUACACCUCAAUGUCCUCUTT-3′), a Sox9 siRNA duplex (sense: 5′- GACUCACAUCUCUCCUAAUTT-3′, anti-sense: 5′- AUUAGGAGAGAUGUGAGUCTT-3′) and a scrambled siRNA-duplex (indicated by “Mock”) were used (Eurogentec). ATDC5 cells were seeded at 25,000 cells/cm^2^ and transfection with siRNAs (100 nM for p65 and 50 nM for Sox9) was performed using ICAfectin 442 (Eurogentec) according to manufacturers' protocol. Cells were cultured for 2 days before chondrogenesis was initiated.

### hBMSC isolation and culture

Human bone marrow stem cells (hBMSCs) were obtained from residual iliac crest bone marrow aspirate from young, genetically healthy individuals undergoing spinal surgery. The Maastricht University Medical Centre institutional policy on the use of residual human surgical material specifically states that no informed consent is needed in the case of residual surgical material. However an approval from the institutional Medical Ethical Committee (MEC) for the use of this material is required. The MEC approved this study and assigned approval ID: MEC 08-4-056. Human BMSCs were isolated from the aspirate using Ficoll Paque (Amersham). Proliferation medium consisted of DMEM high-glucose (Invitrogen,), 10% FCS (ES-grade), 1% antibiotic/antimycotic and 1% NEAA. Passage 5 cells were plated at 30,000 cells/cm^2^ and chondrogenesis was initiated the next day by changing to differentiation medium (proliferation medium supplemented with 1% ITS (Invitrogen), 50 µg/ml L-ascorbic acid-2-phosphate (Sigma) and 1 ng/ml TGFβ3 (R&D)) [53]. In experiments for Figure 5B the proliferation medium was changed with proliferation medium. Medium was changed every 2 days. TLCK (Acros) was used to inhibit NF-κB/p65 activation and LPS (Sigma) was used as NF-κB/p65 activator.

### Chicken periosteum agarose culture (cPAC)

Fertilized eggs of Dekalb white chickens ('t Anker, Ochten, the Netherlands) were placed in a polyhatch incubator (Brisnea) at 39.2°C and at relative humidity of 40%. At embryonic day 16, embryos were removed and sacrificed by rapid decapitation. Incubation period corresponded to embyos at Hamburger and Hamilton stage 42. Periosteum was dissected from tibiae using aseptic techniques. Periostea were embedded in 1% low-melting agarose/0.9% NaCl using procedures described before [23], [54]. Proliferation medium (DMEM/F12, 10% FCS, 1% antibiotic/antimycotic, 1% NEAA) was added and incubated overnight at 37°C/5% CO2. The next day, medium was changed with proliferation medium supplemented with or without LPS (Sigma). For the differentiation control, medium was changed to differentiation medium (proliferation medium supplemented with 1% ITS, 50 µg/ml L-ascorbic acid-2-phosphate, 10 ng/ml TGFβ3) with or without TLCK (Acros). Medium was changed every 2 days. Ethical approval by the institutional animal ethical committee was waived for these experiments as institutional regulations state that no approval from an animal ethical committee is needed to perform embryonic chicken experiments.

### Mouse growth plates

The growth plates were isolated from tibias of 6 weeks old C57BL/6 mice. These were surplus wildtype mice from another unrelated experiment. This experiment was approved by the Maastricht University animal ethical committee (DEC) and assigned approval ID: DEC 2008-042. The tibia's were isolated and fixated in formalin. The growth plates were separated from the rest of the tibia and decalcified in 0.5 M EDTA pH 7.8 for 2 weeks. EDTA was refreshed every 2 days. Growth plates were dehydrated and embedded in paraffin. Five micrometer sections were cut and positioned on Superfrost Plus slides for IHC.

### RT-qPCR

Total RNA was extracted with TRIzol (Invitrogen). Quantity and purity of extracted RNA were determined by UV-spectrometry (Nanodrop, Thermo Scientific). DNA-free total RNA was reverse transcribed to cDNA using standard procedures and random hexamer priming.

Real time quantitative PCR (RT-qPCR) was performed using Mesagreen qPCR mastermix plus for SYBR Green (Eurogentec) and an Applied Biosystems ABI PRISM 7300 Sequence Detection System for amplification with the following profile: initial denaturation 10 minutes at 95°C, followed by 40 cycles of amplification (15 seconds at 95°C and 1 minute at 60°C), followed by a dissociation curve. Data were analyzed using the standard curve method and relative quantification of mRNA expression was normalized to a housekeeping mRNA. Primer sequences are depicted in Table S1.

### Immunoblotting

Cells were lysed in RIPA buffer (150 mM NaCl, 1% NP-40, 0.5% Sodium deoxycholate, 0.1% SDS, 50 mM Tris pH 8.0, 5.0 mM EDTA pH 8.0, 0.5 mM dithiothreitol and 1 mM phenylmethylsulfonylfluoride). Nuclear extracts were prepared by lysing cells in a buffer containing 20 mM HEPES (pH 7.8), 20 mM KCl, 4 mM MgCl_2_, 0.2 mM EDTA (pH 8.0), 1 mM dithiotreitol, 0.2 mM sodiumvanadate, 0.4 mM phenylmethylsulfonylfluoride, 0.3 mg/ml leupeptin and 0.2 mM sodiumfluoride). Nuclei were separated from cytoplasm by centrifugation (16.100× g) and the nuclear pellet was lysed in RIPA buffer. Extracts were sonicated and protein concentrations were determined using the BCA method (Sigma). Polypeptides were separated by SDS-PAGE and transferred to nitrocellulose membranes by electroblotting. Primary antibodies for immunodetection were anti-Col2A1 (Southern Biotech), anti-Col10A1 (Calbiochem), anti-Sox9 (Abcam), anti-COX-2 (Cayman), anti-iNOS (Abcam), anti-p65 (Santa Cruz Biotechnologies), anti-RunX2 (MBL) and anti-α-Tubulin (Sigma). Bound primary antibodies were detected with immunoglobulins conjugated with HRP (DakoCytomation) and visualized by ECL. ECL signals were quantified using Biorad Quantity One 4.6.7 software and relative differences, corrected for background and housekeeper, were determined as compared to control conditions or t = 0.

### (Immuno)histochemistry

Chicken PAC samples were dehydrated following standard procedures and embedded in paraffin. Tissue sections were cut at 5 µm, deparaffinized and rehydrated using standard protocols. Proteoglycans were stained with Safranin-O (0.1%) and counterstained with Fast Green (0.1%). Stained sections were dehydrated and mounted in Histomount (Thermo Shandon). For Sox9, p65, iNOS and COX-2 expression in 6-weeks old mouse growth plate and cPAC, sections were deparaffinized and antigen retrieval was performed by incubation in boiling citrate buffer (1.8 mM citric acid and 8.2 mM tri-sodium citrate) for 30 minutes. For Col2A1 detection in cPAC, sections were digested with 0.4% hyaluronidase (Sigma) for 30 minutes at 37°C. Endogenous peroxidase activity was inactivated and sections were blocked with 10% normal sheep serum. Primary antibodies were: anti-Sox9 (sc-166505; Santa Cruz Biotechnology), anti-p65 (sc-372; Santa Cruz Biotechnology), anti-COX-2 (610203; BD Transduction Laboratories), anti-iNOS (ab3523; Abcam) and anti-Col2A1 (II-II6B3; Developmental Studies Hybridoma Bank). Similar antibody concentrations were used for negative controls; mouse IgG1 (Dako) for COX-2 and Col2A1, mouse IgG2a (Dako) for Sox9, normal rabbit serum for iNOS and anti-p65 blocking peptide (sc-372P; Santa Cruz Biotechnologies) for p65. After washing in PBS-T, bound antibodies were detected with HRP-labelled secondary antibodies (Dako, EnVision+ System-HRP labelled Polymer). For visualisation, DAB substrate (Dako) was used. Stained sections were counterstained with Mayer's Hematoxylin (Dako), dehydrated and mounted in Histomount as described above.

### Statistics

In the Figures, bars represent average value of 3 individual experiments (performed in triplicate; 3×3 samples) and error bars represent mean ± SEM. Statistical significance (p<0.05) was determined by unpaired two-tailed student t-tests using Graphpad PRISM 5.0.

## Supporting Information

Figure S1 **NF-κB/p65 nuclear translocation during early ATDC5 differentiation.**
(DOC)

Figure S2 ***In silico*** **scan for NF-κB/p65 transcription factor binding sites in the Sox9 promoter.**
(DOC)

Figure S3 **Chondrogenic differentiation is enhanced by stimulation of NF-κB/p65 activity.**
(DOC)

Figure S4 **NF-κB signaling during chondrogenic differentiation of hBMSCs.**
(DOC)

Figure S5 **Connection between early NF-κB/p65 activation and growth factor expression in ATDC5 chondrogenic differentiation.**
(DOC)

Table S1 **DNA oligo sequences for RT-qPCR.**
(DOC)
