# Supplementary material for: Activation of NF-κB/p65 Facilitates Early Chondrogenic Differentiation during Endochondral Ossification
Source: PLoS One. 2012 Mar 12;7(3):e33467. doi: 10.1371/journal.pone.0033467 (PMC3299787; doi:10.1371/journal.pone.0033467)
Supplement: Figure S1 — NF-κB/p65 nuclear translocation during early ATDC5 differentiation. (DOC) [file pone.0033467.s001.doc]

**
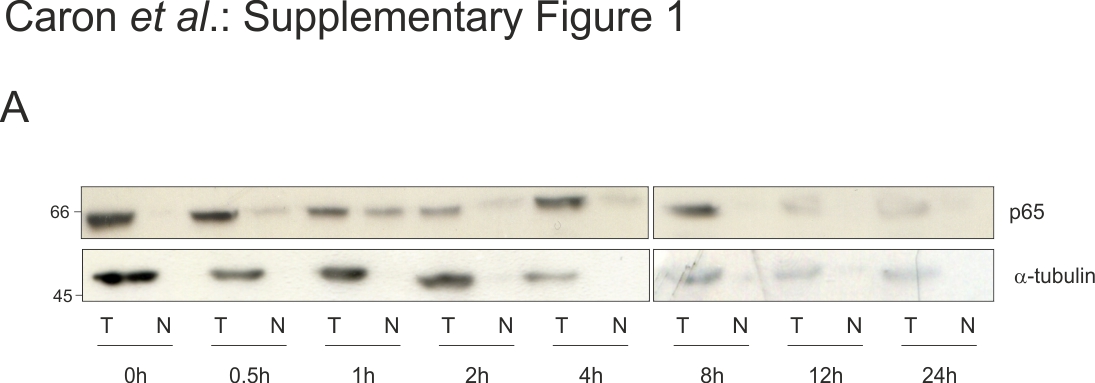
**

**Figure S1: NF-κB/p65 nuclear translocation during early ATDC5 differentiation.**

**A:** In addition to Figure 1A, nuclear translocation of p65 was besides at 0, 0.5, 1 and 2 hours in differentiation also determined at 4, 8, 12 and 24 hours in differentiation of ATDC5 cells. Total extract (T) and nuclear (N) fractions were isolated. Cytoplasmic marker: α-tubulin. NF-κB/p65 is transiently activated at 0.5 – 4 hours in differentiation.
