# Supplementary material for: Activation of NF-κB/p65 Facilitates Early Chondrogenic Differentiation during Endochondral Ossification
Source: PLoS One. 2012 Mar 12;7(3):e33467. doi: 10.1371/journal.pone.0033467 (PMC3299787; doi:10.1371/journal.pone.0033467)
Supplement: Figure S2 — In silico scan for NF-κB/p65 transcription factor binding sites in the Sox9 promoter. (DOC) [file pone.0033467.s002.doc]

Homo sapiens -1000 ------------------TTGGTCTTTTACAAACCAAGTGACC-GGCCTGGGCCTCGCGGCCCG-GGACAGCCGCATTGGCAAACTTCTATCTCTCAAAGCCAGAGCAGT
Macaca mulatta -1000 GCCGTGGTGCCCATTTGTTTGGCCTTTTGCAAACCAAGTGACC-GGCCTGGGCCTCGCGGCCCG-GGACAGCCGCATTGGCAAACTTCGATCTCTCAAAGCCAGAGCAGT
Gorilla gorilla -1000 ----------CCATTTGTTTGGTCTTTTACAAACCAAGTGACC-GGCCTGGGCCTCGCGGCCCG-GGACAGCCNCATTGGCAAACTTCGATCTCTCAAAGCCAGAGCAGC
Pan troglodytes -1000 ------------------TTGGTCTTTTACAAACCAAGTGACC-GGCCTGGGCCTCGCGGCCCG-GGACAGCCGCATTGGCAAACTTCTATCTCTCAAAGCCAGAGCAGT
Mus musculus -1000 ---------C-----------GC------------------CC---------------GGCCC------TGCCACCTTTGCAAACTTCGC--CCTCAACCCCGGAGTAGT
Sus scrofa -1000 TG-------CGCATTTGTTTGGCCTTTTTCAAACC-AGTGACC-GGCCTCAGCCTCGCGGCCCA-GAACAGCCACATTTGCAAAGTTCGGTTTCTCAAAGCCAGAGCAGT
Cavia porcellus -1000 -------------------TGGAGTTTGACAAACCAAGTGACCCTTCCTCGGCCTCTGGGCGCG-GGAGGGCCACACTCGCAAAGTTCTGTC--CC-AAGCTAACGCAGT
Tursiops truncatus -1000 ------------------------------AAACCAAGGGACT-GGCCTCAGCCTCGCGGCCCA-GGACAGCCACATTCGCAAAGTTCGACTTCTCAAAGCCAGACCAGT
Oryctolagus cuniculus -1000 ---------CCCAACTGTGTGGCCTTTTACAAA------------------G----CCAGCCCG-TGACCGACGCTCTCCCAAAAGTCGGTCTCCGAAAGCCAGCGCGGT
Canis familiaris -1000 ---------------------GTCCCTTGCAAGCCG-GAGCC-CTTTG-CAAGCT--CTTCCCAACGGCGACCCTGCTGACA--GTGTGGACTCGGCCGGCCGG-G-AGG


Homo sapiens -910 TAGCAAACTCTCCCCCAGACAGGGCGACTCGGCTGACGTTTTT-GACCCGGCCAGG-AGGCAAAGACCAAAACGTCAGAGCAGT---AGCCCTGTTACTGAGGAGCGTC
Macaca mulatta -892 -TAGCAAACTCTCCC-CAGGCAGGACGACTCGGCTGACGTTTCT-GACTCGACCAGG-AGGCAAAGACCAAAACGTCAGAGCAGT---AGCCCTGTTACTGAGGAGCGTC
Gorilla gorilla -902 -TAGCAAACTCTCCC-CAGACAGGGCGACTCGGCTGACGTTTTT-GACCCGGCCAGG-AGGCAAAGACCAAAACGTCAGAGCAGT---AGCCCTGTTACTGAGGAGCGTC
Pan troglodytes -910 -TAGCAAACTCTCCC-CAGACAGGGCGACTCGGCTGACGTTTTT-GACCCGGCCAGG-AGGCAAAGACCAAAACGTCAGAGCAGT---AGCCCTGTTACTGAGGAGCGTC
Mus musculus -951 TTTGCAAACTC-----CAG---TGGCGAGCCTGACGGTGTTTGT-GACTCAGTCAGG-AGGCAAGAAGCAGAACTCTAAAGTAAT---AATCCTGACTACGAGGGGCGTC
Sus scrofa -900 -ACGCAAACTCTTTT-CTG---CGGCAGCTCTGCTGTCGT-TCT-GACTCGGCCAG--AGGTGAAAAGCAGAACTTTAAAGCACT---TGCCCTGTCACTGAGGAGCGTC
Cavia porcellus -913 -TTGCAGGCTCTTTC-CAG---GGCCAACGCTGCTGACGTTTCTGGACTCGGCCAGG-AGGCAAAGAGCAGAACGTGAAAGCAGCGGCAACCCTGTCCCCCAGGGGCGTC
Tursiops truncatus -922 -ATGCAAACTCTTTC-CAG---GGGCAACTCTGCTGACGT-TCT-GATTCGACCAG--AAGTGAAAAGCAGAACCTCCGAGCAGT---CGCCCTGTCACGGAGGAGGGTC
Oryctolagus cuniculus -922 -TGGCAAAGTTTGC-GGGAGCAGC-TGCAGCCGCCGCCGCCTCT-GACTCGGCCGCGCCGGTGAGGAGCGCAGCGTCAACACAGT---CGCCCAAAGGCTCAGAAGCTTA
Canis familiaris -920 T-G-AAAA-----------GCAGAGCGTCAAAGCAGTTGCCTGT-CACTAAGCCGCG-TGGCGGGGTGGGG-GCTCTGGGGT---GGGGGT-----------GGAGGGCC


Homo sapiens -806 GGCAGGGTC------GCGGG----TA--GAGGGGGCTGGAGAATGACTTGTCAGAGCTCAAGGTCGA-TGTGGCG--CGGGG-CGGC-CTCGAGAGCGCCGGGCTCCTGC
Macaca mulatta -789 GGCGGGGTC------GC--------------GGGACTGGAGAAAGACTTGTCAGAGCTCAAGGTCGG-TGTGGCG--CGGGG-CGGC-CCCGAGAGCGCGGGGATCCTGC
Gorilla gorilla -799 GGCAGGGTC------GCGGG----TA--GAGGGGGCTGGAGAATGACTTGTCAGAGCTCAAGGTCGA-TGTGGCG--CGGGG-CGGC-CTCGAGAGCGCCGGGCTCCTGC
Pan troglodytes -807 GGCAGGGTC------GCGGG----TA--GAGGGGACTGGAGAATGACTTGTCAGAGCTCAAGGTCGA-TGTGGCG--CGGGG-CGGC-CTCGAGAGCGCCGGGCTCCTGC
Mus musculus -854 A---GAGTA-----------CTGG---A--G-GGGCCGGGAAAGGACTTGTCA--GTTCAAGGTCGG-CGTGGCCCACGGGACCGGC-CGCAAGAGCCGGGGA---CTGC
Sus scrofa -802 GGCAGGGTGGGAGTCGCGGGG-GGTGGGGTGGGGGCTGGAGAAAGACTTGTCGGAGTTCAAGGTCGG-CGAGGCG--CGGGA-CGGC-CCCGCGAGCTCGCAACTACCGC
Cavia porcellus -809 GGCGGGGTG------GGGG------AC--TGCGGGCCGGAGAAGGACTCGTA-AAGTTCAAGGTTGA-GGTGGCGCGCGGGC-CGGC-CCCG---------CA---CCGC
Tursiops truncatus -824 GGCGGGGTG------GGGGGCTGGGGAGGGGGCGGCTGGAGAAGGACTTGTCAGAGTTCAAGGTCGG-TGAGGCGCGCGGGA-CGGCTCCCGCGAGCGCGGGGCTGCGTC
Oryctolagus cuniculus -819 GGCGGGGAG------AGGGG-----CCA--GGGGTCCCTGGGAGGACTTGTAGGAGTTCAAGGTCGGGGGTGGCT--CGGAG-AGGC-CGCGGCAGCGCGAGGCTACAGC
Canis familiaris -840 TG-GGGGCG------GGGGG--GG--GG--GGGTGCTGGAGTAGGACTTGTCAGAGTTCAAGGTCGC-TGCGGCG--CGGGA-CGGC-TCCGGG-GC--T-AA---CTGC


Homo sapiens -713 GT---GGCCACGGCCGCCGCTGC------CAACCTTCGCGG-GGACTTAGCTTTGCTTTCCATTGACTCCC-TTTGCAAAA--------GCGCAGCAGAATC-CTGACCA
Macaca mulatta -704 GT---GGCCACGGCCGCCGCTGC------CAACCTTCGCGG-GGACTTAGCTTTGCTTTCCATTGACTCCC-TTTGCAAAA--------GCGCAGCAGAATC-CTGACCA
Gorilla gorilla -706 GT---GGCCACGGCCGCCGCTGC------CAACCTTCGCAG-GGACTTAGCTTTGCTTTCCATTGACTCCC-TTTGCAAAA--------GCGCAGCAGAATC-CTGACCA
Pan troglodytes -714 GT---GGCCACGGCCGCCGCTGC------CAACCTTCGCGG-GGACTTAGCTTTGCTTTCCATTGACTCCC-TTTGCAAAA--------GCGCAGCAGAATC-CTGACCA
Mus musculus -771 GC---GGTCCCT------GCCGC------CAGCCTTTGCCTGGTGCTCGGCTTTGGTTTTCATTGATTCCCCACTGCGGGA--------GCGCAGCAGAAGC-TCCAGTC
Sus scrofa -698 GT---TGCCGCG------ACCTC------CAGCCTGAGCCG-GGACCTAGCTTTGGTTTTCATTGATTCCC-TTTGCAAGA--------GCGCAGCCGAATC-CCAACCG
Cavia porcellus -729 A------CCGCT---GC----GC------CTGCCTCCGCCG-GGGCTCAGCTCTGCTTTTCATTGATTCCC-TTCGCGACA--------GCGCAGCAGAATC-CCGACCG
Tursiops truncatus -722 GT---CGCTGCG------GCCGC------CAGCCTGAGCGG-GGACCTCGCTGTGGTTTTCATTGATTCCC-TTTGCAGGA--------GCGCAGCCGAATC-CCGACCA
Oryctolagus cuniculus -726 GTCCCGGCCGCCGCCGCCGCCGCCGCCTCCCGCCCTGGCGG-GGACGTAGCTTCGGTTTCCATTGATTCCC-CGTGCAGGG--------GCGCAGCAGAATC-CCCCCCA
Canis familiaris -755 G------CCGCG------GCCGC------CGGCCTCCGCGG-GGACCCAGCTTCGGTTTCCATTGATTCCCCTTTGCAAGAGCGAGCGAGCGCACCCGAACCCCCGACCC


Homo sapiens -623 GCCGCACCAGCCCCGGCGAACCCGAGCATGTTA-ATCTATTTATATGGATTATTACGGAGGAACAGCGGGCGTTGAGTCACCAAAACA-TTTGCTTC-AAAAGACTATTT
Macaca mulatta -614 GCCGCACCAGCCCCGGCGAACCCGAGCATGTTA-ATCTATTTATGTGGATTATTACGGAGGAACAGCGGGCGTTGAGTCACCAAAACA-TTTGCTTC-AAAAGACTATTT
Gorilla gorilla -616 GCCGCACCAGCCCCGGCGAACCCGAGCATGTTA-ATCTATTTATATGGATTATTACGGAGGAACAGCGGGCGTTGAGTCACCAAAACA-TTTGCTTC-AAAAGACTATTT
Pan troglodytes -624 GCCACACCAGCCCCGGCGAACCCGAGCATGTTA-ATCTATTTATATGGATTATTACGGAGGAACAGCGGGCGTTGAGTCACCAAAACA-TTTGCTTC-AAAAGACTATTT
Mus musculus -685 ACCACACCAGCTTCGTTGAACCAGAGCGCGTTA-ATCTATTTATATGGATTATTACAGAG-AACAGCGGGCGTTGAGTCACCCAAACA-TTTGCTTCCAAAAGACCATTT
Sus scrofa -614 GCCGCACCAGTCCCTGTGAAGCAGAGCATGTTA-ATCTGTTTATATGGATTATTACAGAGGAACAGCGGGTGTTGAGTCACCAAAACA-TTTGTTTC-AAAAGACAATTT
Cavia porcellus -649 GCCGCACCAGCCCCGGCGAACCAGAGCATGTTA-ATCTATTTATATGGATTATTACGGAGGAACAGCGGGCGTTGAGTCACCAAAACG-TTTGCTTC-CAAAGATCATTT
Tursiops truncatus -638 GCCGCACCAGCCCCGGCGAACCAGAGCATGTTAAATCTATTTATATGGATTATTACCGAGGAACGGCGGGCGTTGAGTCACTAAAACT-TTTGCTTC-GCAATACAATTT
Oryctolagus cuniculus -627 GCCGCGCGGGCCCCGGCGAAGCGGAGCATGTTA-ATCTCTTTATATGGATTATTACCGAGGAACAGCGGGCGTTGAGTCACCAAAACA-TTTGCTTC-CCAAGACCATTT
Canis familiaris -664 GCCGCACCAGCCCGGGCGAACCGGAGCGTGCTA-ATCTATTTATACGGATTATTACGGAGGACCGGCGGGCGTTGAGTCGCCAAAATAAGTTGCTTC-AAAAGACCATTT


Homo sapiens -516 CTAAGCACTTTTGCAGGCAGGCAGGCTCGCTCCAGGCGCGTAAACTCGGCTACGCATTA----AGAAGCGGCTGCT----TTTCGAATACTGCAAACTCC---AGCTAAG
Macaca mulatta -507 CTAAGCACTTTTGCAGGCAGG----CAGGCCCCAGGCGCGTAAACTCGGCTACACATTA----AGAAGCGGCTGCT----TTTCGAATACTACAAACTCC---AGCTAAG
Gorilla gorilla -509 CTAAGCACTTTTGCAGGCAGGCAGGCTTGCTCCAGGCGCGTAAACTCGGCTACGCATTA----AGAAGCGGCTGCT----TTTCGAATACTGCAAACTCC---AGCTAAG
Pan troglodytes -517 CTAAGCACTTTTGCAGGCAGGCAGGCTCGCTCCAGGCGCGTAAACTCGGCTACGCATTA----AGAAGCGGCTGCT----TTTCGAATACTGCAAACTCC---AGCTAAG
Mus musculus -578 CTAAGCACTTTTTTTGGAA-GCCGGCAGACTCCAGGCGCAGAAGCCCAGCTCCGCTTTG----ACGAGCAGCTGTTGCAATTTCCATTGCTGTAAACGCC---AGCGAAG
Sus scrofa -507 CTAAGCACTTTCGGAGG-AGG----CAAGCTCCAGGCGCGGAAACTGGGCTACGCTTTA----AGGAGCTACAGTT----TTTTGAATACTGCAAACTCCCCTGGCTTAG
Cavia porcellus -542 CTAAGCACTTTTGGAAGCGAGCA-GCAGGCTCCAGGCGCAGAAAG-GGACTACACATTT----AGGAGCGGCTGCT----TTTCCAATATTGAAAACTCC---AGCGAAG
Tursiops truncatus -530 CTAAGCGCTTTCGGCGG-AGG----CCGGCTCCAGGCGCGGAAACTGGGCTATGATTTAAGTCAGGAGCGACAGCT----TTTCGAATATGGCAAACTCC---GACTAAG
Oryctolagus cuniculus -520 CTAAGCACTTTTGGAGGCGGGCAGGCAGGCTCCAGGCGCGCAAACCCGGCTGCGCAGGA----AGGAGCGACTGCT----TTTCGAACACTGCGAACTCC---GGTGAAG
Canis familiaris -556 CTAAGCACTTTCGGAGG-GGG----CAGGCTCCGGGCGCAGGCACGGGGCTGCGCGTTC----CGGAGCGGGCGCT----TTCCGAATGCTACAGACTCC---AGCCAAG


Homo sapiens -417 TCCCCGGTGCCGCGGAGAGAGCAGTGA-AAAGAAATG-T-CGGAGGTGGG--G---GTAGATCCTAGTCTAGACACACACAC----------------------TTGCGC
Macaca mulatta -412 TCCCCTGTGCCGCGGAGAGAGCAGTGA-AAATAAATG-T-CGGAGGTGGG--G---TTAGATCCTAGTCTAGACATACACAC----------------------TTGCGC
Gorilla gorilla -410 TCCCCGGTGCCGCGGAGAGAGCAGTGA-AAAGAAATG-T-CGGAGGTGGG--G---GTAGATCCTAGTCTAGACACACACAC----------------------TTGCG-
Pan troglodytes -418 TCCCCGGTGCCGCGGAGAGAGCAGTGA-AAAGAAATG-T-CGGAGGTGGG--G---GTAGATCCTAGTCTAGACACACACAC----------------------TTGCGC
Mus musculus -476 TCCCGGGTACCACGGAGACAGCATCGA-AAAGTGGGGGT-GGGGGGTTGT--G---GAGGGTCCTAGTCTAGACACGCTCGCGTGCACGCGCACACACACACACACACAC
Sus scrofa -410 TCCCCAAGACCGCGGAGAAAGCAGTGA-ACAGAAACGCT-TGGGGGTGGG--G---ATAGATCCTAGTCTACACACACACAC----------------------ACACAC
Cavia porcellus -445 TCCC-GGTGCCGCCAAGAGACCAGTGA-AAAGAAGTG-T-C-GAGGTGGG--G---GCAGATCTCAGTCTAGACACACGCCCGCGA--GTGCACGCATGCAC--ACACAC
Tursiops truncatus -432 TCCCCGGGACCGCGGAGAAAACAACGA-ACAGAAATGCT-TGGGGGTGGG--G---AGAGATCCTAGTCTACACACACACAC----------------------ACACAC
Oryctolagus cuniculus -421 TCCTCCGGACCGCAGAGAGAACAAGGGGAAGGGAGTTAT-CAGGGGTGAGCGGGCCGTGGCTCCTAGTCTAGACACACG------------------------C--GCGC
Canis familiaris -462 CCTCGAGGACCGTGCAGAAAGCAGGGG-AAAGAAATGCTCCGGGGGTGGG--G---GCAGAGCCTAGTCTAGACACGCGCACGCGGGCGCGCGCGCACACACAC--AGAC


Homo sapiens -337 G--------CACACACACACACACACACA--------C----------A--AGATTCGCGCGGAGAA-GG-CACTAAAATTCTGGCATTCCGAGAGTAC--GACAAACTT
Macaca mulatta -332 GCG------CGCGCGCACACACACACACA--------C----------A-AAGATTCGCGCGGAGAA-GT-CACTAAAATTCTGGCATTCCGAGAGTTC--GACAAACTT
Gorilla gorilla -331 ---------CACACACACACACACACACA--------C--------------GATTCGCGCGA---A-GG-CACTAAAATTCTGGCATTCCGAGAGTAC--GACAAACTT
Pan troglodytes -338 G--------CACACACACACA--CACACA--------C----------A--AGATTCGCGCGGAGAA-GG-CACTAAAATTCTGGCATTCCGAGAGTAC--GACAAACTT
Mus musculus -373 ACACATACACACACACACACACACACACACACACACACA------CACA-TCGGTTCACACGGAGAC-CG-TTCCAAAACTGTGACATTCCGAGAGTAGAGAGCAAACTT
Sus scrofa -329 ACA--------CACACACGCACACGCGCG--------CG------CGCA-GAGATTCGCGCGGAGAC-GG-TACCG-AATTCTGACATTATGAGAGTAC--AGCAAACTT
Cavia porcellus -349 ACA------CACACACACACACACACACACACGTGCGCG------CGCT-AAGTTTCTTGCGAACAC-TG-TACCAAAATTCTGACATT-----TGTAC--GGCA-ACTT
Tursiops truncatus -351 ACA------CACACACACACACACACACG------GGCG------CGCA-AAGATTCGCGCGGAGAC-GG-CACCG-AATTCTGACATTACCAGAGCAC--GGCAAACTT
Oryctolagus cuniculus -338 G--------CACAGGCACACACGCGCAAA--------C--------------TACCCGCGCGGAGAAAGGCCCCCGACATGCTGACATTCCGAGAGTGC--GGCAAACTT
Canis familiaris -360 ACACA--CACATAGACACGCGCGCACACGCGCACACGCAGGCAGGCACGCAAAATTCGTGCAGAGAC-GG-CACCACACTTCTGACATTACGAGAGCAC--GGCAGACTT


Homo sapiens -259 ACACACTTGGAAGTCCCGGGTCCCCCGCCTTCCCCGCAGCACCCCCCGCC--CC-C---CCACC-CTACCGTCCGCCCTTTGGCTGCGATCCCCTCCCCTCTCCTCCCCT
Macaca mulatta -251 ACACACTTGGAAGTCCCGGGTCCCCCGCCTTTCCCGCAGCACCCCCCGCC--CCCC---CTTCC-CGACCGTCCACCCTTCGGCTGCGCTCCCCTCCCCTCTCCTCCCCT
Gorilla gorilla -259 ACACACTTGGAAGTCCCGGGTCCCCCGCCTTCCCCGCAGCACCCCCCGCC--CC-C---CCATC-CTACCGTCCACCCTTTGGCTGCGATCCCCTCCCCTCTCCTCCCCT
Pan troglodytes -262 ACACACTTGGAAGTCCCGGGTCCCCCGCCTTCCCCGCAGCACCCCCCGCCCCCCCC---CCACC-CTACCGTCCGCCCTTTGGCTGCGATCCCCTCCCCTCTCCTCCCCT
Mus musculus -272 ACACACTCGGACGTCCCGGGTCCCCCGCTTGCCCCGCGCCCCCCTCCAAGTC-CCC---TCACC-CCACCATCCACCCTCTGGCTGAGCTCCCCTCCCTTCTCCTCCCCT
Sus scrofa -247 ACACACTTGTAGGTCCCGGGTCC----------CCGCAGCACCCCCCC---CCCCA---AAACC--C---GCCGGACCAGCGGCAGTGCTCCCCTCCCCGCTCATCCCCT
Cavia porcellus -262 ACATATTTGGACGTCCCGGGTCCCCCGCCTTC-CCGCAGCACTCCCCCCACCCTCTACCCCCTC-CCACCATCCACCCTTGGGCTGCGGTTCCCTCCCCTCTCCTCCCCT
Tursiops truncatus -265 ACACACTTGGACGTCCCGGGTCCCCCGCTTTCCCCGCAGCACCCCACC--CCCCCA---ACCCC-CCACCGGCGGACCAGCGGCTGCGCTCCCCTCCCCGCTCCGCCCCT
Oryctolagus cuniculus -260 ACACACTTGGACGTCCCGGGTCCCCCGCCTTCCCCGCGGCACCCCCACCACCACC-CCC-CACCACCACCATCCACCCTTGGGCTGCGCTCCCT-----TCCCCTCCCCT
Canis familiaris -256 ACACACTTGGACGTCCCGGGTCCCCCGCCTTCCCCGCTGCACCCCCACCTCCCCCCCTCCCAAC-CCCGCGCCAGACCAGAGGCAGCGCTCCCCTCCCCGCTCCTCCCCT

Homo sapiens -156 CCCGCCTCGTCACCCA-GCCCAGTGCCACAATCCTCCTC--CCTCC---CCAA-AATCGGGTCCAATCAGCTGCCTGCCAA-CCCTG------GGACTGCTG----TGCT
Macaca mulatta -147 CCCGTCTCGTCACCCA-GCCCAGTGCCGCAATCCTCCTC--CCTCC---CCAA-AATCGGGTCCAATCAGCTGCCTG----------------GGACTGCTG----TGCT
Gorilla gorilla -156 CCTGTCTCGTCACCCA-GCCCAGTGCCACAATCCTCCTC--CCTCC---CCAA-AATCGGGTCCAATCAGCTGCCTGCCAA-CCCTG------GGACTGCTG----TGCT
Pan troglodytes -156 CCCGTCTCGTCACCCA-GCCCAGTGCCACAATCCTCCTC--CCTCC---CCAA-AATCGGGTCCAATCAGCTGCCTGCCAA-CCCTG------GGACTGCTG----TGCT
Mus musculus -167 CCTGTCTCGTCACCCA-ACCCGGAGCCACAATCCTCCCCAGCCCCCCTTCCAA-AATCCGGTCCAATCAGCGACTTGCCAA-CACTGA-----TGACTCAAGAGCTAGCC
Sus scrofa -158 CCCCTCTAGTCACCCA-GCCCAGAGCCACAATCCTCCCC--CCCCC---CCACCAGACTGCGCCAATCAGCTGCCTGACAA-CCCCC------GAAATGCCG----TGCT
Cavia porcellus -154 CCGGTCTCTTCACCCA-GCCCAGAGCCACAATCCTCCTC--CCCAC---TCCA-AATCGGGCCCAATGAGCTGCTTGTCAG-CC--A------GGACTGCCG----GGCT
Tursiops truncatus -161 CCCCTCTCAACACCCT-GCCCAGCGCCACAATCCTCCTC--CCCC----CCG--AAGTGGATCCAATCAGCTGCCTGCCAAGCCCTGAAACCTGAAATGCCG----TGCT
Oryctolagus cuniculus -157 CCCGTCTCGTCACCCA-GCCCAGTACCACCATCCTTCTC--CTCCC---CTAT-ACCCCGGCCCAATCAGCTGCCTGCCAA-CCCCG------AGACTGCCG----CGCT
Canis familiaris -147 CGCCTTTCGTCACCCAAGCCCGGAGCCT----------T--AC-CCG---CAA-AATCCAGTCCAATCAGCTGCCTGCCCA-CCCCT------GGAATGCCG----GGCC

Homo sapiens -64 GTGATTGGCGG-GTGGCTCTAAGGTGA-GGCGGAGTATTTATTAAAGAGACCCTGGGCTGGGAGTT
Macaca mulatta -64 GTGATTGGCGG-GTGGCTCTAAGGTGA-GGAGGAGTATTTATTAAAGAGACCCTGGGCTGGGAGTT
Gorilla gorilla -64 GTGATTGGCGG-GTGGCTCTAAGGTGA-GGCGGAGTATTTATTAAAGAGACCCTGGGCTGGGAGTT
Pan troglodytes -64 GTGATTGGCGG-GTGGCTCTAAGGTGA-GGCGGAGTATTTATTAAAGAGACCCTGGGTTGGGAGTT
Mus musculus -65 GTGATTGGCCCGAGGTATCTAACGTGAAGGAGGAGTATTTATTA—-GAGACCCTGAGCTGGAAGTC
Sus scrofa -65 GTGATTGGCTAGAGGTCCCTAAGGTGA-GGGGGTGTATTTATTAAAGAGCCCCTGGGCTGGGAGTT
Cavia porcellus -64 GTGATTGGCTGGACGTCTCTAAGGTGA-GG-GGAGTATTTATTGAGAAGACCCCGGGCTGGGAGTC
Tursiops truncatus -64 GTGATTGGCTG-ACGTCTCTAAGGTGA-GGGGGTGTATTTATTAAAGAGCCGCTGGGCTGGGAGTT
Oryctolagus cuniculus -65 GTGATTGGCCGGGCGTCCCTAAGGTGA-GTGGGTGTATTTATTAAAGCGACCCCGGGCTGGGAGTT
Canis familiaris -65 GTGATTGGCCGGCCGTCTCTAAGGTGA-GGCGCAGTAGTTATTAAAGAGCCCCGGGGCCGGGAGTC

**Figure S2: *In silico* scan for NF-κB/p65 transcription factor binding sites in the Sox9 promoter.**

Genomatix’s Gene2Promoter software (www.genomatix.de) was used to screen Sox9-promoters (-1000 bp region) from 10 different vertebrates for putative NF-κB transcription factor binding sites, based on the consensus binding sequence for NF-κB: GGGRNNYYCC (in which R = purine, N = any nucleotide and Y = pyrimidine) (1). A multiple nucleotide sequence alignment was generated by the T-Coffee multiple sequence alignment program (http://www.tcoffee.org/) and >50% nucleotide homology is depicted in black boxes generated by the Boxshade server v3.21 (http://www.ch.embnet.org/software/BOX_form.html). Evolutionary conserved putative NF-κB transcription factor binding sites are marked in grey boxes. In addition to the functional NF-κB transcription factor binding sites found by Ushita and colleagues (2), two other putative evolutionary conserved NF-κB transcription factor binding sites were detected. Ensembl references to used promoter sequences are *Homo sapiens*: GRCh37:17:70116161:70117161:1, *Macaca mulatta:* MMUL_1:16:67356426:67357426:1, *Gorilla gorilla*: gorGor3:5:1129374:11298374:-1, *Pan troglodytes:* CHIMP2.1:17:71547493:71548493:1, *Mus musculus*: NCBIM37:11:112642524:112643524:1, *Sus scrofa:* Sscrofa9:12:6872241:6871241:-1, *Cavia porcellus*: cavPor3:scaffold_3:74364652:74365652:1, *Tursiops truncates:* turTru1:scaffold_92737:81033:82033:1, *Oryctolagus caniculus*: oryCun2:19:55819664:55820664:1, *Canis familiaris*: BROADD2:9:11347542:11348542:1

References

1. Quandt, K., Frech, K., Karas, H., Wingender, E., and Werner, T. (1995) *Nucleic Acids Res* 23(23), 4878-4884

2. Ushita, M., Saito, T., Ikeda, T., Yano, F., Higashikawa, A., Ogata, N., Chung, U., Nakamura, K., and Kawaguchi, H. (2009) *Osteoarthritis and Cartilage* 17: 1065-1075
