# Supplementary material for: Activation of NF-κB/p65 Facilitates Early Chondrogenic Differentiation during Endochondral Ossification
Source: PLoS One. 2012 Mar 12;7(3):e33467. doi: 10.1371/journal.pone.0033467 (PMC3299787; doi:10.1371/journal.pone.0033467)
Supplement: Figure S3 — Chondrogenic differentiation is enhanced by stimulation of NF-κB/p65 activity. (DOC) [file pone.0033467.s003.doc]

**
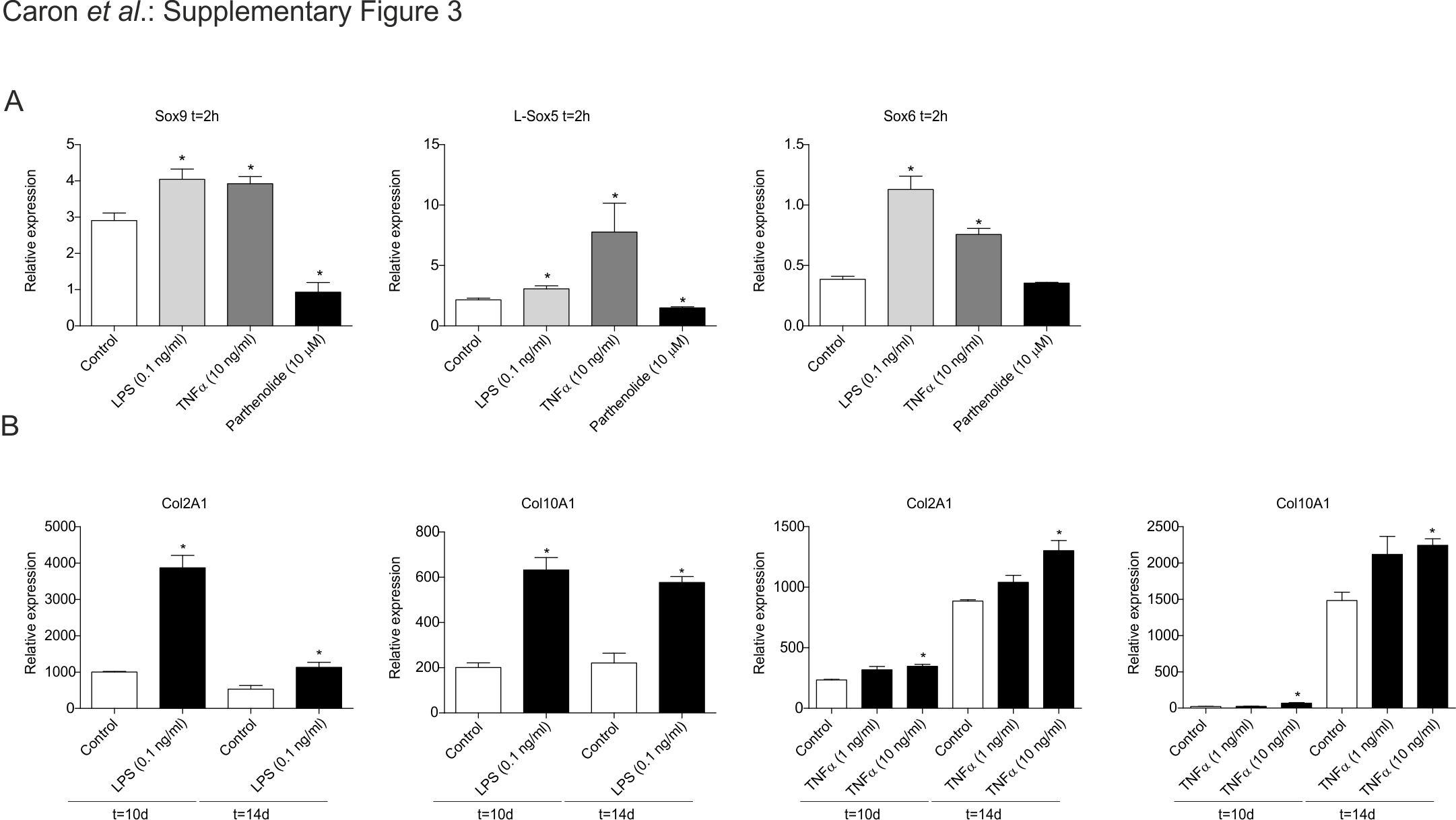
**

**Figure S3: Chondrogenic differentiation is enhanced by stimulation of NF-κB/p65 activity.**

**A:** ATDC5 was cultured for 2 hours in the absence or presence of LPS (0.1 ng/ml), TNFα (10 ng/ml) or Parthenolide (10 µM) and mRNA expression of the “Sox-trio”; Sox9, L-Sox5 and Sox6 was determined. Both LPS and TNFα resulted in increased Sox9 expression confirming data in Figure 3B and Pathenolide treatment resulted in decreased Sox9 expression (Figure 2B). Although Sox6 expression was generally low, similar responsivity to NF-κB activation or inhibition were obtained for L-Sox5 and Sox6 expression, supporting the suggestion that Sox9 might function in early chondrogenic differentiation in conjunction with L-Sox5 and Sox6. **B:** Gene expression data from Figure 3D. ATDC5 was cultured for 10 or 14 days in the absence or presence of LPS (0.1 ng/ml) during the first 24 hours. Expression of Col2A1 and Col10A1 mRNAs was determined by RT-qPCR. Right panel is similar as for LPS but with 1 or 10 ng/ml TNFα. Both LPS and TNFα resulted in increased levels of chondrogenic differentiation, confirming protein expression shown on Figure 3D. Asterisks in graphs indicate a significant (p < 0.05) change in expression compared to control or Mock conditions.
