# Supplementary material for: Activation of NF-κB/p65 Facilitates Early Chondrogenic Differentiation during Endochondral Ossification
Source: PLoS One. 2012 Mar 12;7(3):e33467. doi: 10.1371/journal.pone.0033467 (PMC3299787; doi:10.1371/journal.pone.0033467)
Supplement: Figure S4 — NF-κB signaling during chondrogenic differentiation of hBMSCs. (DOC) [file pone.0033467.s004.doc]

**
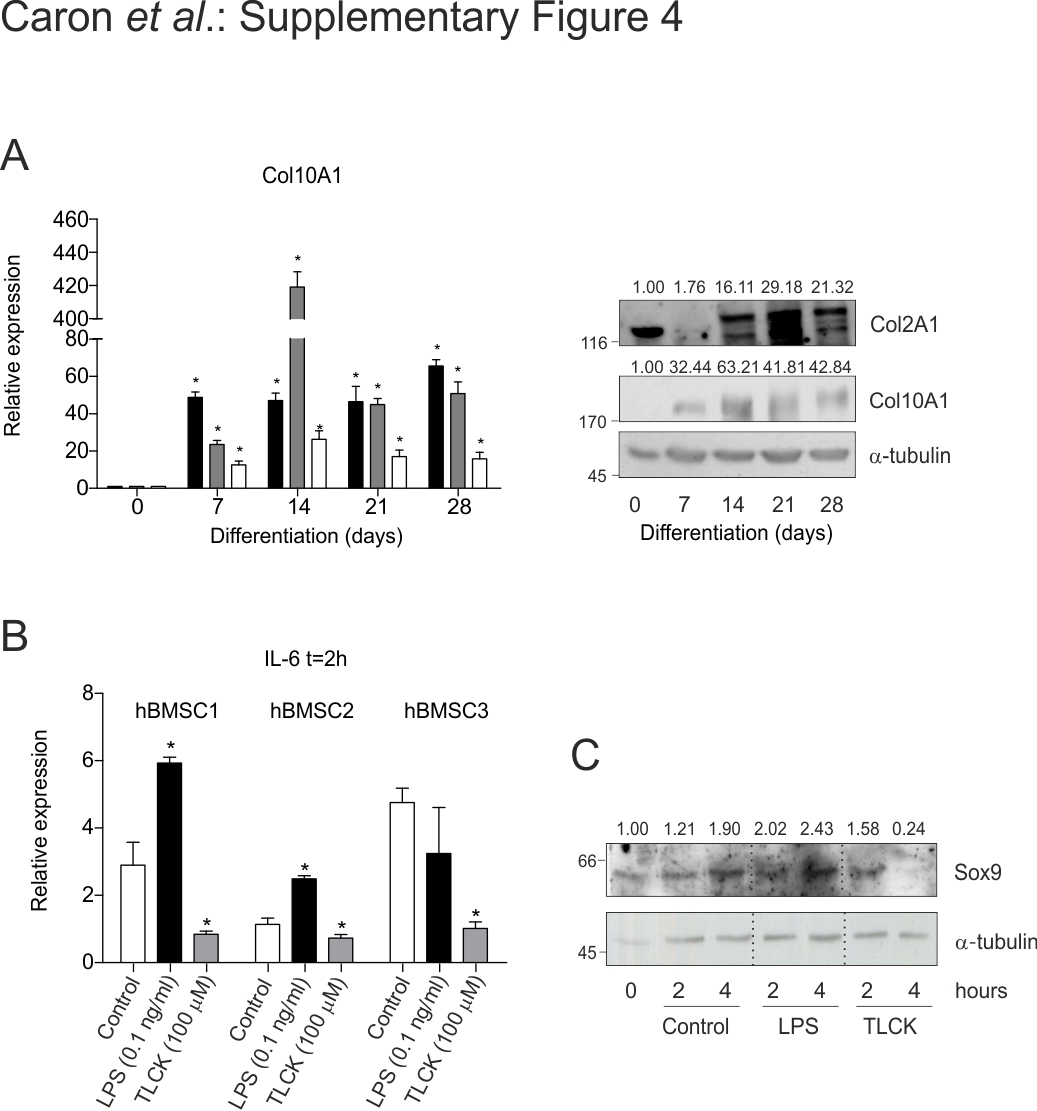
**

**Figure S4: NF-κB signaling during chondrogenic differentiation of hBMSCs.**

Human bone marrow stem cells from three individuals (hBMSC1/2/3) were differentiated into the chondrocyte lineage using monolayer culture. **A:** Col10A1 mRNA expression in hBMSCs was assessed at day 0, 7, 14, 21 and 28 (left panel; normalized to 28S rRNA). Right panel shows a representative example of Col2A1 and Col10A1 protein expression at same time points. **B:** hBMSCs were differentiated for 4 hours in the presence of LPS (black bars) or TLCK (grey bars) from onset of differentiation. White bars represent the control condition. Expression of IL-6 mRNA was determined by RT-qPCR. **C:** In similar samples from (B) Sox9 protein expression at 2 and 4 hours in differentiation was determined by immunoblotting.
