# Supplementary material for: Activation of NF-κB/p65 Facilitates Early Chondrogenic Differentiation during Endochondral Ossification
Source: PLoS One. 2012 Mar 12;7(3):e33467. doi: 10.1371/journal.pone.0033467 (PMC3299787; doi:10.1371/journal.pone.0033467)
Supplement: Figure S5 — Connection between early NF-κB/p65 activation and growth factor expression in ATDC5 chondrogenic differentiation. (DOC) [file pone.0033467.s005.doc]

**
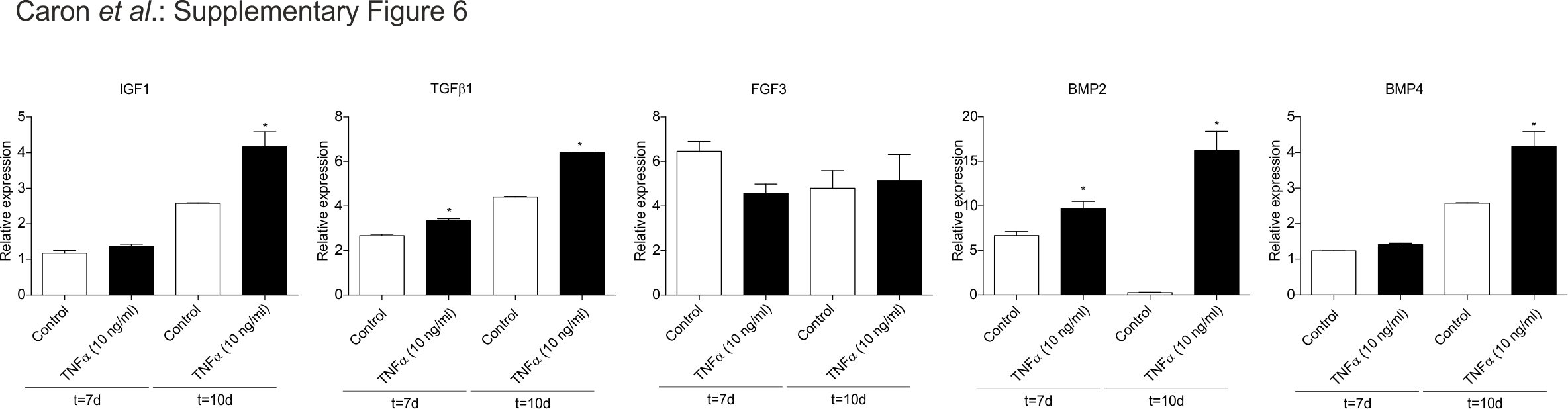
**

**Figure S5: Connection between early NF-κB/p65 activation and growth factor expression in ATDC5 chondrogenic differentiation.**

To investigate the connection between the early NF-κB/p65 activation and expression of chondrogenic differentiation markers (see Figure 3D) at day 10 and 14, we determined the expression of multiple important chondrogenic growth factors. Addition of NF-κB-activator TNFα (10ng/ml) for the first 24 hours resulted in increased expression of the chondrogenic growth factors at day 7 and/or 10 in differentiation (except for FGF3), consistent with the data from Figure 3.
