# Supplementary material for: Activation of NF-κB/p65 Facilitates Early Chondrogenic Differentiation during Endochondral Ossification
Source: PLoS One. 2012 Mar 12;7(3):e33467. doi: 10.1371/journal.pone.0033467 (PMC3299787; doi:10.1371/journal.pone.0033467)
Supplement: Table S1 — DNA oligo sequences for RT-qPCR. (DOC) [file pone.0033467.s006.doc]

**Table S1. DNA oligo sequences for RT-qPCR.**


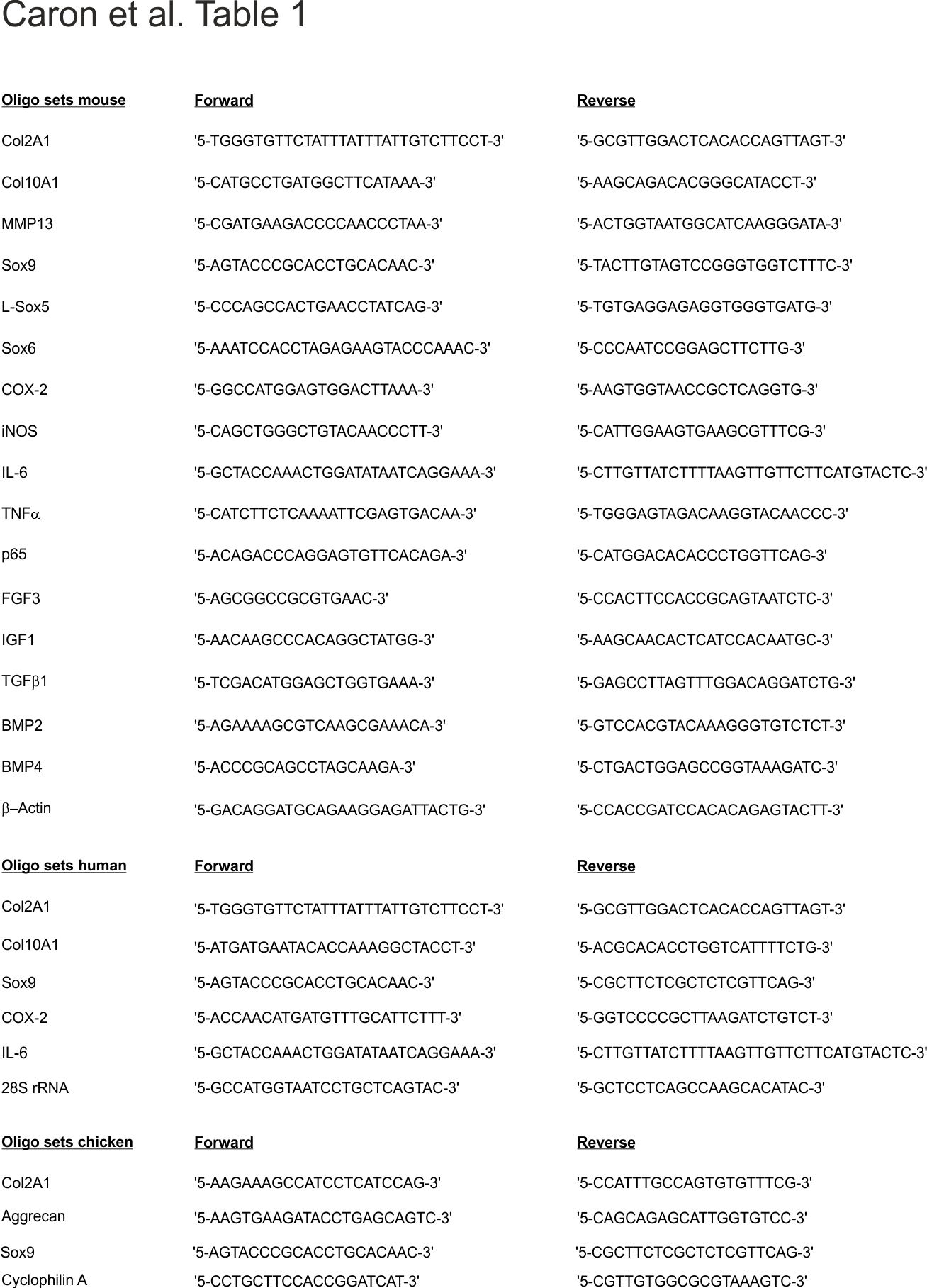


Forward and reverse oligo sequences (5’-3’) used for RT-qPCR are listed for mouse, human and chicken.
